# Cytoprotective Effects of Antioxidant Peptides from Red Californian Worm (*Eisenia foetida*) Hydrolysate on Differentiated Caco-2 Cells

**DOI:** 10.3390/nu16213654

**Published:** 2024-10-27

**Authors:** Yhoan S. Gaviria, José E. Zapata, Diego Miedes, Amparo Alegría, Antonio Cilla

**Affiliations:** 1 Nutrition and Food Technology Group, Faculty of Pharmaceutical and Food Sciences, University of Antioquia, Medellin 050010, Colombia; yhoan.gaviria@udea.edu.co (Y.S.G.); edgar.zapata@udea.edu.co (J.E.Z.); 2 Nutrition and Food Science Area, Faculty of Pharmacy and Food Sciences, University of Valencia, Avda. Vicente Andrés Estellés s/n, 46100 Burjassot, Valencia, Spain; diego.miedes@uv.es (D.M.); amparo.alegria@uv.es (A.A.)

**Keywords:** oxidative stress, cytoprotective effect, antioxidant peptide, molecular docking, red Californian worm (*Eisenia foetida*)

## Abstract

Background/Objectives: When prooxidants outweigh antioxidants, oxidative stress can occur, causing an accumulation of reactive oxygen species (ROS). This process can lead to cellular damage and plays a role in the development of numerous health conditions. This study aimed to investigate the cytoprotective effects on differentiated Caco-2 cells of hydrolysates derived from the red Californian worm (WH) and their fractions, identify the peptides responsible for this effect, and elucidate the mechanisms involved. Methods: The WH was obtained through hydrolysis with Alcalase 2.4 L and subsequently fractionated to two fractions (F > 3 kDa and F < 3 kDa) using a ceramic membrane with a molecular weight cutoff of 3 kDa. The peptides found in the F < 3 kDa fraction, demonstrating the highest cytoprotective activity, were then sequenced via liquid chromatography-mass spectrometry analysis (LC-MS/MS), and molecular docking was conducted to elucidate the underlying antioxidant mechanisms. Results: The hydrolysate of *Eisenia foetida* and its F < 3 kDa fraction exhibited no cytotoxicity, protected the cells from H_2_O_2_-induced oxidative stress (50% increase viability), preserved cell viability by restoring their redox status (ROS: 20% decrease, and glutathione (GSH): recovered to basal control levels) and cell cycle distribution, and decreased apoptosis (16%). Twenty-eight peptides were identified, with five showing antioxidant activity through stable interactions with myeloperoxidase (MPO) and Kelch-like ECH-associated protein 1 (Keap-1), KPEDWDDR being the peptide that presented the highest affinity with both molecules (−7.9 and −8.8 kCal/mol, respectively). Conclusions: These results highlight the WH as a potential source of bioactive peptides for the management of oxidative stress.

## 1. Introduction

ROS are naturally produced as byproducts during cellular metabolism and are essential for processes like signal transduction and maintaining homeostasis. However, oxidative stress arises when there is a disruption in the balance between pro-oxidants and antioxidants. This imbalance leads to the buildup of ROS, causing cellular damage and contributing to a range of health issues such as cancer, cardiovascular and neurological diseases, hypertension, rheumatoid arthritis, autoimmune disorders, inflammation, degenerative conditions associated with aging, and diabetes mellitus [1]. Antioxidants can mitigate oxidation by donating a hydrogen atom or an electron to form free radicals, thereby terminating oxidative chain reactions and removing initiating or intermediary radicals. This action may involve scavenging singlet oxygen species or deactivating metal catalysts [2]. Several enzymes contribute to oxidative damage in the human body. Myeloperoxidase (MPO) interacts with hydrogen peroxide (H_2_O_2_), released by phagocytic cells when they encounter foreign particles, forming a highly reactive enzyme–substrate complex [3]. Elevated MPO activity has been linked to various pathological conditions, increasing the risk of oxidative stress, such as in infections, inflammatory disorders like rheumatoid arthritis, and ischemia/reperfusion injury [4]. Conversely, antioxidant peptides can mitigate oxidative stress through multiple mechanisms, with the Keap1/Nrf2-ARE signaling pathway being a key regulator in the body’s endogenous oxidative defense system [5].

Antioxidant peptides have been obtained from different agro-industrial substrates such as red tilapia [2], *Morchella* protein [6], and red tilapia scales [7], among others. A protein source with great growth is the worm, particularly *Eisenia foetida*, which represent a significant proportion of the invertebrate biomass in diverse soil types, accounting for 60–80% of the overall biomass found in soils [8]. Its broad role in improving soil aeration, moisture regulation, nutrient cycling, and in general, the soil structure, underscores their vital contribution to the physical and chemical dynamics of terrestrial ecosystems [9]. Commonly cultivated using underutilized resources such as livestock manure, household scraps, and agro-industrial residues, worms are primarily utilized in the production of bio-compost [10]. They are recognized for providing biologically and pharmacologically active substances, which are employed in the treatment of a wide range of diseases [11]. Historically, *E. foetida* has been employed in therapeutic practices in Chinese and Hindu traditions [12]. Recent studies indicate that *E. foetida* is a substantial protein source [13], a crucial nutrient for a range of cellular functions, ranging from structural support to metabolic and regulatory processes [14]. Moreover, they are substrates for different hydrolyses, including enzymatic, leading to the generation of bioactive peptides and other advantageous compounds. This hydrolytic process involves peptide bond cleavage via water and enzymatic or chemical catalysis, altering molecular properties such as reduced molecular weight, increased charge, and exposure of hydrophobic groups [15]. Bioactive peptides not only deliver nitrogen and essential amino acids but also offer additional physiological advantages beyond their core nutritional functions [16]. Antioxidant peptides demonstrate notable structure–activity relationships, where specific amino acid sequences, molecular weight, and hydrophobicity play essential roles in their radical scavenging capacity. For instance, peptides such as LKPGN and LQP, derived from Antarctic krill (*Euphausia superba*) hydrolysates, exhibit strong antioxidant activity due to their smaller molecular size, which enhances their ability to interact with free radicals [17]. Similarly, peptides like EDIVCW (MMP-4) from monkfish (*Lophius litulon*), and GEYGFE from sturgeon (*Acipenser baerii*) cartilage, show a high scavenging activity against DPPH and hydroxyl radicals, likely due to the presence of hydrophobic and aromatic amino acids, which improve their ability to stabilize radicals [18,19]. These peptides improve the activity of key antioxidant enzymes, such as superoxide dismutase (SOD) and glutathione peroxidase (GSH-Px), while simultaneously decreasing oxidative markers like reactive oxygen species (ROS) and malondialdehyde (MDA) [20]. Their protective mechanisms, including mitochondrial stabilization and apoptosis inhibition, highlight their potential in therapeutic applications aimed at combating oxidative stress [18].

On the other hand, Caco-2 cells that are fully differentiated could be an effective and valuable model for assessing how enterocytes respond to different oxidative stresses [21]. Previous studies have shown the protective antioxidant effects of peptides from enzymatic hydrolysis against oxidative stress in differentiated Caco-2 cells. Zhang et al. [6], reported the protein hydrolysate from *Morchella esculenta* (MPH) at concentrations of 62.5–250 µg/mL decreased H_2_O_2_-induced cytotoxicity in Caco-2 cells. It exhibited protective effects by lowering ROS and malondialdehyde (MDA) levels, strengthening antioxidant defenses, and activating the Nrf2 signaling pathway. Additionally, it has been reported that MPH can inhibit H_2_O_2_-induced apoptosis by restoring the integrity of the mitochondrial membrane potential [6]. Likewise, treatment with red tilapia (*Oreochromis* spp.) viscera hydrolysates (RTVH) at a concentration of 0.1 mg/mL and 0.25 mg/mL of the <1 kDa fraction demonstrated no cytotoxic effects and did not alter the redox state, cell cycle distribution, or induce cell death in differentiated Caco-2 cells [2]. Different sources of hydrolysates and peptides have been evaluated, such as fish proteins [2,22], edible mushrooms such as *Morchella esculenta* [6], and *Agaricus bisporus* [23], soybean [24], and sheep plasma [25] in Caco-2 cells, to determine the cytoprotective effect. On the other hand, the bioactivity of worm (*Amynthas arenulus*) peptides obtained by enzymatic hydrolysis in liver carcinoma cells (HepG2) and healthy mouse fibroblasts (L929) have demonstrated its antioxidant capacity, ability to safeguard cells from oxidative stress, and its potential to enhance immune lymphocyte proliferation [26]. However, no studies have been conducted to date on the cytotoxic and cytoprotective effects of hydrolysates from red Californian worm (*E. foetida*) in cell cultures.

In this context, molecular docking has gained prominence as a vital technique for predicting how peptides interact with target molecules, determining the strength of these interactions, and identifying the key amino acid residues responsible [27]. This technique is commonly applied to analyze binding patterns between small molecule ligands and receptors by calculating binding affinities, identifying binding sites, and providing detailed information on molecular interactions [28]. Therefore, the subsequent phase will focus on assessing the bioactivity of red Californian worm peptides in a biologically relevant system, specifically, differentiated Caco-2 cells, to further explore their potential health-related functions. Recognizing the need to explore alternatives for managing oxidative stress in the human body, this research aimed to investigate the protective effect against oxidative stress induced by hydrolysates from *E. foetida* and its fractions in differentiated Caco-2 cells, identify peptides responsible for cytoprotective effects, and elucidate the mechanisms by molecular interaction.

## 2. Methods and Materials

### 2.1. Materials and Reagents

The *Eisenia fetida* were supplied by the company “Lombrices de Tenjo” (Cundinamarca, Colombia; 4°52′11″ N 74°08′38″ W) and were delivered through the feeding substrate. The 3-(4,5-dimethylthiazol-2-yl)-2,5-diphenylthiazolium bromide (MTT) (CAS No.: 298-93-1), hydrogen peroxide (H_2_O_2_) (CAS No.: 7722-84-1), 5(6)-Carboxy-2′,7′-dichlorofluorescein diacetate (DCFDA) (CAS No.: 111843-78-8), and propidium iodide (PI) (CAS No.: 25535-16-4), Annexin V (UNSPSC Code: 12352207) were sourced from Sigma-Aldrich (St. Louis, MO, USA). The 5-Chloromethylfluorescein diacetate (CMFDA green) and fluorochrome Fluo-3/AM were obtained from Thermo Fisher Scientific (Waltham, MA, USA). Corning, located in New York, NY, USA, supplied the phosphate-buffered saline (PBS) (Ref: 21-040-CM). Non-essential amino acids (Ref: 11140050), antibiotic solution (penicillin-streptomycin) (Ref: 15140122), Dulbecco’s modified eagle medium (DMEM+ GlutaMAX™) (Ref: 10569010), fetal bovine serum (FBS) (Ref: A5670801), trypsin-EDTA solution (containing 2.5 g/L trypsin and 0.2 g/L EDTA) (Ref: 25200056), antifungal agent (Fungizone) (Ref: 15290018), and HEPES (Ref: 11560496), were procured from Gibco (Scotland, UK). Alcalase 2.4 L was obtained from Novo Nordisk Co., located in Bagsvaerd, Denmark.

### 2.2. Enzymatic Hydrolysis and Fractionation by Tangential Ultrafiltration

The enzymatic hydrolysis was performed using a Brunswick bioreactor (Eppendorf, Hamburg, Germany) with a capacity of 6000 milliliters, as reported by previous investigations [29]. The crude protein from *E. foetida* (CRW) was prepared at a concentration of 20 g/L, with the pH adjusted to 8.5 using 2 N NaOH. Alcalase 2.4L^®^ was then added at 0.75 U/g protein (enzyme-to-substrate ratio), and the mixture was stirred continuously at 240 rpm while maintaining a temperature of 45 °C. The hydrolysis process was carried out for 67 min. The Alcalase 2.4L^®^ enzyme was selected and optimized in previous studies, because it had the highest catalytic activity to hydrolyze the protein [29]. After hydrolysis, the hydrolysate was processed using a tangential ultrafiltration system (Standex Electronics, Inc., Fairfield, OH, USA) equipped with a ZrO_2_ ceramic membrane on an Al_2_O_3_ support (Tami Inc., Nyons, France), with a molecular weight cutoff of 3 kDa, as previously described [30]. This fractionation yielded two distinct fractions as follows: F > 3 kDa, containing molecules larger than 3 kDa; and F < 3 kDa, comprising those smaller than 3 kDa.

### 2.3. In Vitro Cytoprotection

#### 2.3.1. Cell Culture

Human adenocarcinoma cells (Caco-2) ((HTB-38) HT-29) were purchased from the American Type Culture Collection (ATCC) (Rockville, MD, USA). Cells were used between passages 6–16. The cells were maintained and grown in Dulbecco’s Modified Eagle Medium+ GlutaMAX™ (4.5 g/L glucose), supplemented with 10% (*v*/*v*) FBS, 1% (*v*/*v*) non-essential amino acids, 1% (*v*/*v*) HEPES, 1% (*v*/*v*) penicillin-streptomycin solution, and 0.2% (*v*/*v*) fungizone, and incubated (37 °C/5% CO_2_/95% relative humidity) [2]. Caco-2 cells were inoculated in 96- and 24-well plates (Costar Corp., Washington, DC, USA) at a cell density of 3 × 10⁴ cells/cm^2^, and the culture medium was refreshed every two days. Optimal cell differentiation was observed starting from day 7 after seeding (722 ± 88.7 Ω cm^2^) and remained stable through day 12 [2,31]. At 10 days post-seeding, the culture medium was removed, and the cells were pre-incubated for 24 h with the sample or with Dulbecco’s Modified Eagle Medium+ GlutaMAX™ (4.5 g/L glucose), as appropriate.

#### 2.3.2. Cell Viability

To analyze the cytotoxicity of the samples, cells were treated with hydrolysate samples (WH, F < 3 kDa and F > 3 kDa) diluted in DMEM at concentrations ranging from 0.025 to 1 mg/mL, following a review of the literature on hydrolysates from different protein sources [32,33,34]. Cell viability was assessed indirectly using the MTT assay, which measures mitochondrial metabolic rate, as described by Gomez et al. [2]. Using a Synergy H1 microplate reader (BioTek, Agilent, CA, USA), the reduction in MTT to insoluble formazan was measured at 570 nm, with background interference subtracted at 690 nm.

#### 2.3.3. Establishment of H_2_O_2_-Induced Cell Model

Oxidative stress was induced following previous studies conducted by the research group [2,35,36]. After a 24 h pre-incubation with the samples (WH, F > 3 kDa, and F < 3 kDa at 1 mg/mL, that showed the highest viability/protection) or DMEM (stress control), cells were exposed to a 5 mM H_2_O_2_ solution for 2 h under controlled conditions (37 °C/5% CO_2_/95% relative humidity) protected from light. Unstressed cells, incubated with DMEM alone, were used as the cell control.

#### 2.3.4. Determination of Intracellular Reactive Oxygen Species

ROS levels were assessed by detecting fluorescence changes caused by the intracellular oxidation of DCFDA, following the method described by Gómez et al. [2] with slight modifications. The samples were incubated for 24 h, and culture medium was discarded, each well received 1 mL of 10 µM DCFDA solution, and the cells were then incubated at 37 °C for 30 min in complete darkness. The cells were then subjected to oxidative stress as previously described, trypsinized, and collected for flow cytometry analysis (FACS-VerseTm, BD Biosciences, Piscataway, NJ, USA) with an excitation of λ = 495 nm and an emission of λ = 529 nm. For each sample, no fewer than 1 × 10⁴ cells were examined.

#### 2.3.5. Determination of Intracellular Glutathione (GSH)

CMFDA, a fluorescent marker sensitive to GSH, was used for the analysis. The reaction of CMFDA with the thiol group of GSH results in an increase in fluorescence intensity and thus a measure of the intracellular GSH accumulation [37]. The analysis of cellular GSH levels was conducted in accordance with the procedure detailed by Gómez et al. [2]. Cells were incubated in darkness with 1 µM CMFDA at 37 °C for 40 min, and fluorescence intensity was determined by flow cytometry with an excitation of λ = 492 nm and an emission of λ = 516 nm. For each sample, no fewer than 1 × 10⁴ cells were examined.

#### 2.3.6. Intracellular Calcium Content

Intracellular Ca^2+^ concentrations were measured using the fluorochrome Fluo-3/AM [36], with slight adjustments. In brief, suspended and collected cells were placed in a cytometry tube and centrifuged at 1500 rpm for 5 min. Cell pellets were resuspended in 500 µL of PBS, and 10 µL of Fluo-3/AM solution (2 µM, final concentration) were added under dark conditions. The cells were incubated for 40 min at 37 °C, centrifuged at 800 rpm for 5 min, and resuspended in 300 µL of PBS. The fluorescence emitted after Fluo-3/Ca^2+^ binding was measured by flow cytometry with an excitation of λ = 506 nm and an emission of λ = 526 nm. For each sample, no fewer than 1 × 10⁴ cells were examined.

#### 2.3.7. Cell Cycle Analysis

The DNA content of cells is determined by the phase they are in during the cell cycle, and an increase in fluorescence by propidium iodide (PI) staining is proportional to their DNA content. After collecting the cells by centrifugation and washing them with PBS, they were incubated in the dark at 4 °C for 30 min with 200 µg/mL RNase and 20 µg/mL PI. The analysis was performed by quantifying DNA content with flow cytometry with an excitation of λ = 351 nm and an emission of λ = 617 nm, following the methodology described by Gomez et al. [2]. For each sample, no fewer than 1 × 10⁴ cells were examined.

#### 2.3.8. Detection of Apoptosis/Necrosis

Flow cytometry was utilized with double staining to detect phosphatidylserine externalization (early apoptosis) and DNA staining (late apoptosis and necrosis), using Annexin V (excitation λ = 494 nm, emission λ = 519 nm) and PI (excitation λ = 351 nm, emission λ = 617 nm). This method followed the protocol described by Gómez et al. [2]. A 100 µL cell suspension was mixed with 3 µL of Annexin V and 5 µL of PI, then incubated in the dark for 15 min. Subsequently, 400 µL of PBS were added, and the cells were subjected to flow cytometry analysis, analyzing 1 × 10^4^ events for each sample. A two-dimensional method was applied to assess the percentage of viable (Annexin−/PI−), early apoptotic (Annexin+/PI−), late apoptotic (Annexin+/PI+), and necrotic (Annexin−/PI+) cells.

### 2.4. Analysis by Liquid Chromatography-Tandem Mass Spectrometry (LC-MS/MS)

The fraction F < 3 kDa was reconstituted in a solution of 0.1% formic acid (FA) dissolved in water. A 20 mL aliquot of this prepared solution was carefully applied onto an EVO tip (EvoSep, Odense, Denmark), as per the guidelines provided by the manufacturer. For LC-MS/MS analysis, a trapped ion mobility spectrometry, time-of-flight fleX mass spectrometer (Bruker, Billerica, MA, USA) was utilized. The sample, prepared on an Evotip Pure, was introduced into an analytical column (PepSep, 10 cm × 150 µm, 1.5 µm; Evosep) via the Evosep One system. Chromatographic separation was achieved using the 60 SPD method as specified by the manufacturer. Ionization of the eluted peptides occurred in a Captive Spray source at 1700 V and 200 °C, and the analysis was performed in ddaPASEF mode, with the following:

The TIMS settings were configured in custom mode with a 1/K0 range of 0.6–1.6 V·s/cm^2^ and a ramp time of 100 ms, operating at a duty cycle of 100% and a ramp rate of 9.42 Hz. MS averaging was set to 1, and auto-calibration was disabled. For the mass spectrometry settings, the scan range spanned from 100 to 1700 *m*/*z*, with positive ion polarity and the PASEF scan mode enabled. In the MS/MS analysis, four PASEF ramps were performed with a total cycle time of 0.5 s. The charge range was set from 0 (unknown) to 5, and scheduling parameters included a target intensity of 12,500 and an intensity threshold of 1000. Active exclusion was turned on, with no inclusion polygon applied.

System sensitivity was evaluated using 20 ng of digested HELA proteins. A total of 1700 proteins were identified using the 60 SPD gradient. An Uniprot database, named Annalida_17102023, was created with 151,637 entries. MSFragger searches (conducted through FragPipe) were employed to identify enzyme non-specific peptides ranging between 7 and 25 amino acids. Proteomic analysis was performed at the proteomics laboratory of the SCSIE at the University of Valencia, which is a member of the Proteored network.

### 2.5. Molecular Docking Analysis

The three-dimensional peptide structures identified were modeled utilizing Avogadro (Department of Chemistry, University of Pittsburgh) and UCSF Chimera 1.17.3 (National Institutes of Health, University of California, San Francisco). To achieve the most stable energy configuration, the structures were optimized. The interaction between the peptides and target molecules (MPO; PDB ID: 3F9P; Keap-1; PDB ID: 2FLU) was investigated using AutoDock Vina 4.2 (San Diego, CA, USA), and their conformations were refined using a genetic algorithm based on the binding energy minimization principles. PyMOL version 3.0 (San Carlos, CA, USA) was employed to investigate both stereoscopic and planar interactions between the ligand and receptor peptide. The top-ranked docking pose of the purified peptide with the target molecules was determined based on the docking scores and binding energy values.

### 2.6. Data Analysis and Interpretation

The results are presented as the mean ± standard deviation, calculated from five replicates across four separate experiments for each treatment group. Statistical analysis was performed with a 95% confidence interval, utilizing hypothesis testing to compare group means. Fisher’s Least Significant Difference (LSD) test was applied using the Statgraphics Centurion version XVI software. Statistical significance was determined with a *p*-value threshold of 0.05.

## 3. Results and Discussions

### 3.1. Cytotoxicity

The cytotoxic effect of WH and its fractions on differentiated Caco-2 cells is shown in Table 1. It was observed that WH and its fractions did not produce any negative effect on mitochondrial function after 24 h of treatment at any of the tested concentrations compared to the control group (100% viability) (DMEM) (*p* > 0.05). This confirms that they do not exhibit a cytotoxic effect on differentiated Caco-2 cells. The viability (%) of F > 3 kDa and F < 3 kDa is higher compared to WH at the concentration selected for the cytoprotection analysis (1 mg/mL). In the concentration range of 0.025–0.1, the viability of the fractions is lower than WH, however these are not below 100%. The concentrations of WH and its fractions used in the cytotoxicity assay are higher than those reported for fractions under 3 kDa and between 3 and 5 kDa from *A. arenulusen* earthworm hydrolysates. At concentrations of 0.54 and 0.47 mg/mL, the peptides did not exhibit cytotoxicity against normal mouse fibroblast cell lines (L929) [26]. However, a 1 mg/mL treatment of red tilapia (Oreochromis sp.) viscera hydrolysate resulted in a 19% reduction in viability in differentiated Caco-2 cells. This indicates that WH offers certain advantages, as it can be applied at higher concentrations without significantly affecting the activity of normal cells [2]. On the other hand, no cytotoxic effect in Caco-2 cells treated with peptides (1.0 mg/mL) from soybean protein hydrolysate fractions were observed [6].

### 3.2. Cytoprotective Effect

**Figure 1:**
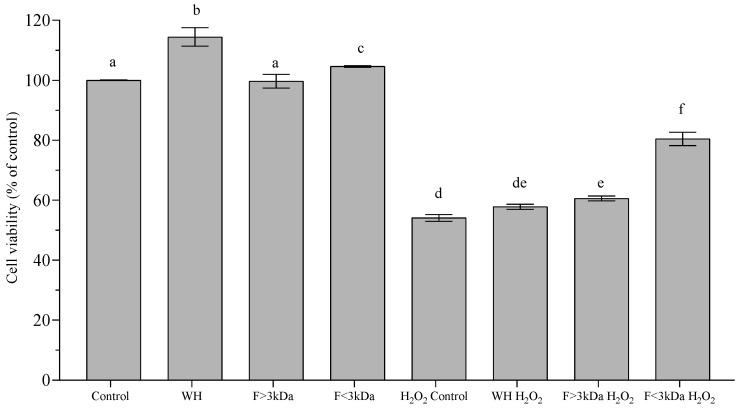
Cytoprotective activity of WH and its fractions (F) on differentiated Caco-2 cells. Results are presented as the mean ± standard deviation, derived from four independent experiments (n = 5). Significant differences (*p* < 0.05) are denoted by different letters (a–f).

The concentration of 1 mg/mL, which showed no cytotoxic effects in the WH and its fractions during the cytotoxicity test, was selected to assess their potential cytoprotective properties against H_2_O_2_-induced oxidative stress. The viability of Caco-2 cells, without and with oxidative stress, is shown in Figure 1. It was observed that treatments with WH and its fractions without exposure to H_2_O_2_ maintained the cell viability of the control. Figure 1 illustrates that H_2_O_2_ treatment led to a significant (*p* < 0.05) decrease in Caco-2 cell viability, with a reduction of around 40% compared to the control group. However, pre-incubation with F < 3 kDa reduced the damage caused by H_2_O_2_, as viability was reduced only by 20%. These findings are consistent with those reported by Gomez et al. [2], who found that the lowest fraction of protein hydrolysates from red tilapia (*Oreochromis* sp.) (1 mg/mL) presented the highest recovery of cell viability. Likewise, soybean protein hydrolysate peptides with lower molecular weight and a higher hydrophobicity showed a higher recovery of cell viability in Caco-2 (96%), because they can efficiently enter cells and interact with the apoptosis-related pathway [37].

**Figure 2:**
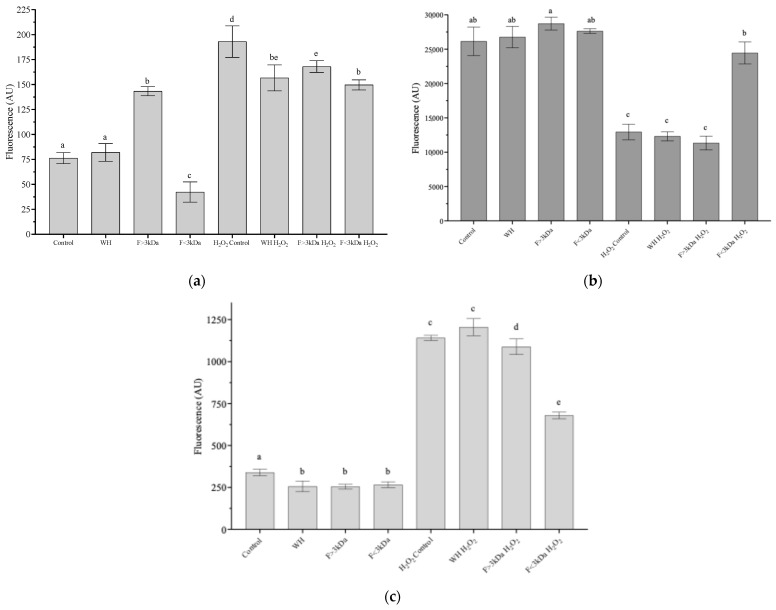
Evaluation of the cytoprotective effect of worm hydrolysate (WH) and its fractions (F) on differentiated Caco-2 cells: (**a**) intracellular ROS; (**b**) intracellular glutathione (GSH); (**c**) intracellular calcium. Results are expressed as the mean ± standard deviation of four independent experiments (n = 5). Significant differences (*p* < 0.05) are denoted by different letters (a–e). AU: Arbitrary units.

The impact on cellular redox status through the accumulation of ROS (Figure 2a) and reduced glutathione (GSH) (Figure 2b), and intracellular calcium levels (Figure 2c) were measured with flow cytometry, widely used due to its high sensitivity and ability to provide information on the redox status of cells [38]. Pre-incubating cells for 24 h with WH or the F < 3 kDa fraction at 1 mg/mL, without exposure to H_2_O_2_, did not cause any significant adverse effects (*p* > 0.05), as illustrated in Figure 2a. Notably, treatment with F < 3 kDa led to a significant 21% reduction in intracellular ROS levels compared to the control (Figure 2a). In contrast, cells exposed to the stress agent exhibited a 2.5-fold increase in ROS concentration compared to unstressed controls. These levels were reduced with F < 3 kDa (20%) to a higher extent (*p* < 0.05) than with F > 3 kDa (12%), but both remained without significant differences with WH (18%). To combat and prevent oxidative stress, cells possess an intracellular defense system that includes both non-enzymatic and enzymatic antioxidant mechanisms. Among the main components of the non-enzymatic system appears to be GSH, in addition to other compounds such as ascorbic acid, tocopherols, and carotenoids [39].

As shown in Figure 2b, intracellular GSH levels remained stable (*p* > 0.05) following pretreatment with WH and its fractions, showing no significant difference compared to the control. However, upon exposure to H_2_O_2_, a notable reduction (*p* < 0.05) in GSH content by 52% was observed. Notably, pretreatment with the F < 3 kDa fraction prior to oxidative stress induction successfully restored GSH levels to those of the non-stressed control group (*p* < 0.05). A key factor in triggering apoptosis is the rise in intracellular calcium levels, which plays a role in destabilizing the mitochondrial membrane potential and promoting the release of pro-apoptotic molecules, such as cytochrome C and endonuclease G [40]. Regarding the intracellular calcium concentration, it was observed that treatments without the stress agent did not present any negative effects compared to the control, (see Figure 2c). When cells were stressed with H_2_O_2_, an increase of up to 4.5 times in the amount of intracellular calcium was observed in the control group, without presenting statistical differences with WH. The fractions F > 3 kDa and F < 3 kDa showed a reduction (*p* < 0.05) in intracellular Ca^2+^ of 10% and 48%, respectively. The lower fraction presents a greater effect (*p* < 0.05), which is consistent with the trend of the MTT and redox state assays. The high cytoprotective capacity of the F < 3 kDa in ROS, GSH, and intracellular Ca^2+^ assays, may be associated with its high content of hydrophobic amino acids (2119.31 mg/100 g) [41]. The concentration of hydrophobic amino acids in antioxidant peptides plays a vital role in defining their physiological impact. Studies have demonstrated that these hydrophobic properties can improve the peptides’ ability to interact with lipid targets or allow them to penetrate certain organs by forming hydrophobic bonds with the lipid bilayer of cell membranes. This phenomenon is beneficial for achieving powerful antioxidant effects [42]. This same trend was observed in antioxidant capacities measured by chemical assays (FRAP: Ferric Reducing Antioxidant Power; TEAC: Trolox Equivalent Antioxidant Capacity; ORAC: Oxygen Radical Absorbance Capacity) [29,30], with the F < 3 kDa showing the highest antioxidant capacity (TEAC: 2404 µequTrolox/g; ORAC: 2041 µequTrolox/g; FRAP: 85 µequTrolox/g). Antioxidants operate through one of the following seven mechanisms, depending on the specific oxidant: (a) neutralizing or scavenging free radicals, (b) chelating metal ions, (c) inhibiting enzymes responsible for free radical production, (d) activating endogenous antioxidant enzymes, (e) preventing lipid peroxidation, (f) shielding DNA from damage, and (g) safeguarding proteins from modification and glycation [43]. The reduction in ROS levels within cells after pretreatment with WH and its fractions, along with their notable capacity to neutralize ABTS* radicals (TEAC) or scavenge oxygen radicals (ORAC), suggests that one of the possible mechanisms of action of the antioxidant peptides present in the WH of *E foetida* and the F < 3 kDa is the sequestration or neutralization of free radicals or ROS. Numerous peptides with antioxidant properties, effective in reducing intracellular reactive oxygen species, have been discovered in protein hydrolysates from various sources, including red tilapia [2], soybeans [24] and fish farming by-products [22]. Along these lines, hydrolysates obtained with Alcalase from chickpea proteins [44], *Morchella esculenta* [6], and soybean [24], have shown a capacity to modulate the activity of key antioxidant enzymes, including glutathione peroxidase and glutathione reductase in Caco-2 cells, which are associated with intracellular GSH production.

### 3.3. Cell Cycle

**Figure 3:**
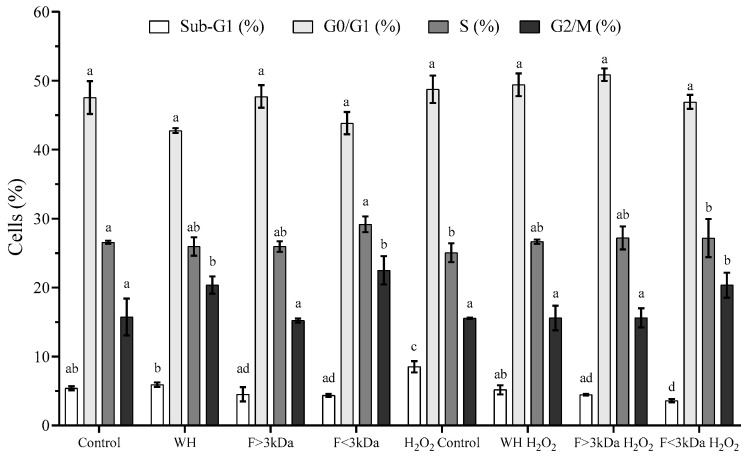
Cell cycle distribution of differentiated Caco-2 cells treated with worm hydrolysate (WH) and its fractions (F). Results are presented as the mean ± standard deviation, derived from four independent experiments (*n* = 5). Different letters (a–d) for the treatments in the same cell cycle phase indicate significant differences (*p* < 0.05).

Figure 3 shows the distribution of the cell cycle in differentiated Caco-2 cells that were pre-incubated with 1 mg/mL of WH and its fractions, both in the absence and presence of H_2_O_2_. Cell cycle analysis showed that WH and its fractions do not produce significant alterations (*p* > 0.05) compared to the control; changes in the Sub-G1, G0/G1, and S phases of the cell cycle were observed, aligning with the findings from other analyzed parameters. Regarding G2/M, WH and F < 3 kDa produced a slight increase in the proportion of cells in this phase. Exposure to H_2_O_2_-induced oxidative stress in differentiated Caco-2 cells led to a significant (*p* < 0.05) 1.6-fold rise in the Sub-G1 cell population, indicating a higher proportion of apoptotic cells compared to the control [40]. Also, a 12% reduction in cells in the S phase, which is vital for DNA replication and centrosome duplication in animal cells, was observed [45]. Pre-treatment of cells with WH and its fractions before oxidative stress induction demonstrated that these samples could return the Sub-G1 cell population to baseline levels (WH and F > 3 kDa) or reduce it by up to 50% in F < 3 kDa. These results indicate that the peptides exert a cytoprotective influence on cell distribution in response to the oxidative stress induced by H_2_O_2_. Protein hydrolysates from animal sources have been reported with similar positive effects on the cell cycle in Caco-2, restoring basal values in the G1 phase as well as in the G2/M phase [2].

### 3.4. Detection of Apoptosis/Necrosis

**Figure 4:**
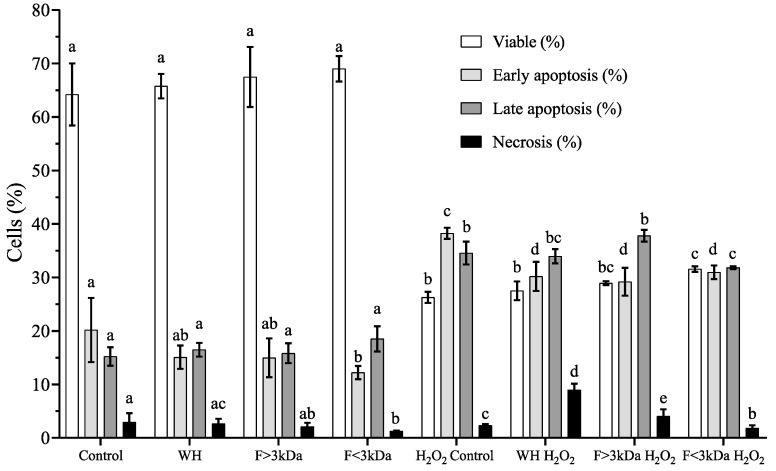
Effect of incubation with WH and its fractions (F) in differentiated Caco-2 cells. Results are presented as the mean ± standard deviation from four independent experiments (n = 5). Significant differences (*p* < 0.05) between treatments within the same cell state are denoted by different letters (a–e).

Oxidative stress may lead to the gradual alteration or breakdown of vital biomolecules, including DNA, proteins, lipids, and carbohydrates. The buildup of damage from reactive oxygen species can disrupt cellular functions, potentially causing cell death through either necrosis (uncontrolled) or apoptosis (regulated) [46]. Figure 4 illustrates the impact of WH and fractions with/without stress agent on cell death in differentiated Caco-2 cells. Treatments without a stress agent did not exhibit any notable adverse effects (*p* > 0.05) with respect to the control. On the other hand, the results indicate that the induction of oxidative stress via H_2_O_2_ leads to a statistically significant (*p* < 0.05) 3-fold reduction in viable cells compared to the control, while the early and late apoptotic cells increased by 46% and 55%, respectively. In stressed cells preincubated with WH and its fractions, the WH and F > 3 kDa did not present statistical differences in viable cell percentages, when compared with the H_2_O_2_-treated control group, while F < 3 kDa showed a 16% recovery. Regarding apoptosis, it was observed that F < 3 kDa reduces early and late apoptosis, while WH and F > 3 kDa only reduce early apoptosis. Pretreatment with F < 3 kDa also manages to reduce necrotic cell values to the levels without H_2_O_2_. In this context, peptides from red tilapia viscera hydrolysates have been reported that fail to prevent cells from entering a state of necrosis, although they did show positive effects on late apoptosis [2]. In this regard, peptides from buffalo casein, applied for 24 h at a concentration of 0.1 μg/mL effectively reverses necrosis-induced cell death, which was triggered by 0.2 mM H_2_O_2_ for 6 h [47]. However, the protective effect diminishes when the peptide concentration is raised to 0.5 μg/mL, resulting in the same level of necrosis in cells as those treated solely with H_2_O_2_. Various agents that induce intracellular oxidative stress, including H_2_O_2_, activate the inherent pathway of apoptosis [46]. All this results in a normal distribution of the cell cycle (S phase) (Figure 3) and the decrease in the sub-G1 phase population, reflecting a decline in apoptosis (Figure 4).

### 3.5. Peptide Identification via LC-MS/MS

Peptide sequence identification was performed on the F < 3 kDa sample since it was the one that presented the most positive results in the cytoprotection assays. Twenty-eight peptides were identified in this fraction, of which five were selected according to their molecular weight, amino acid sequence, and probability. The sequence of the five most likely identified peptides is presented in Table 2. Additionally, Figure S1 displays the mass spectra of the main peptides identified. These peptides have molecular weights (MW) lower than 1500 Da and their length is between 8 and 13 amino acids, aligning with the ultrafiltration process used for their separation. Additionally, the presence of acidic amino acids like aspartic acid (D) and glutamic acid (E), and positively charged amino acids, specifically, lysine (K), which are part of the aforementioned group of hydrophilic amino acids, is observed. This supports the hypothesis that the higher cytoprotective effect observed for the lower MW fraction is due to the presence of these amino acids inside smaller peptides.

### 3.6. Peptide Molecular Docking Analysis

Table 3 presents the results of the molecular docking analysis for the selected peptides, focusing on their interaction with two key molecules involved in regulating oxidative processes, MPO and Keap-1. Concerning the affinity energies, the five selected peptides present relevant affinity values for both molecules, with the peptides KPEDWDDR and SLLDDRLDEK presenting the highest affinity with MPO (−7.3 Kcal/mol), while with Keap-1, the peptides KPEDWDDR and KRVGPGLGEY present the highest affinity (−8.8 and −7.9 Kcal/mol, respectively). In addition, two distinct kinds of π interactions between the peptides and the receptor molecules are observed, as well as the formation of hydrogen bonds at the active sites of MPO and Keap-1.

**Figure 5:**
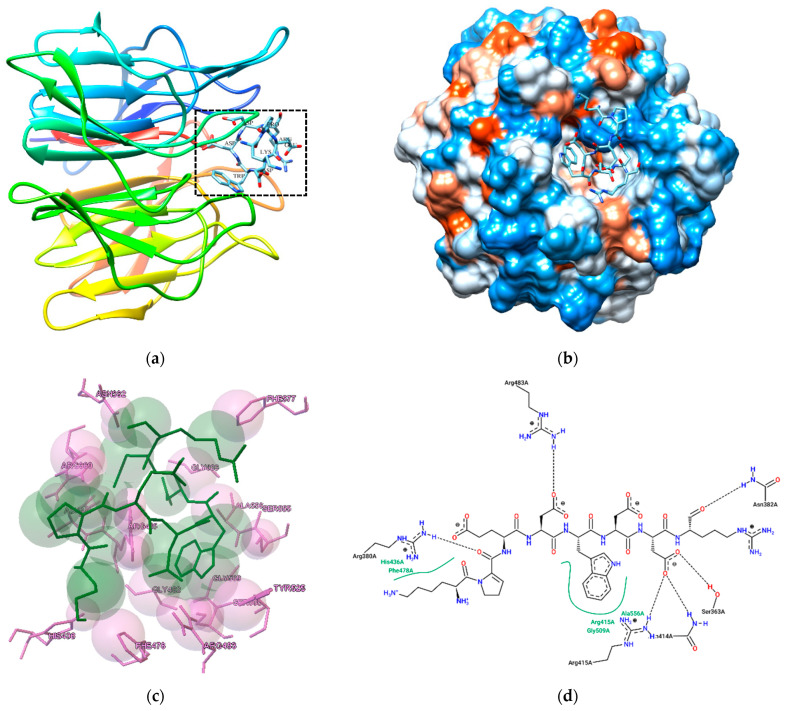
Molecular interaction of the KPEDWDDR peptide-Keap1: (**a**) molecule in quaternary representation; (**b**) surface hydrophobicity diagram; (**c**) charge interactions (pink color: Keap1 molecule; green color: KPEDWDDR peptide); (**d**) 2D interaction diagram.

Figure 5 shows the molecular docking of the peptide KPEDWDDR with Keap-1. Theoretically, inhibitory peptides can inactivate the enzyme by occupying its active sites or blocking entry to the active site cavity [48,49]. In a study, it was found that Nrf2 binds to the Keap1 receptor, forming hydrogen bonds with several residues, including Arg415, Tyr334, Gln530, Ser363, Asn382, Arg380, Arg483, and Ser555 [50]. As depicted in the figure, hydrogen bond interactions were established between the amino acids of the peptide and the specific binding sites Arg380, Asn382, and Arg415 of Keap1, indicating that the peptide directly docks into the active site of Keap1, thereby inhibiting its function. Research has highlighted Arg380 and Arg415 as critical active sites for Keap-1 [51]. On the other hand, oxidative stress can be mitigated by antioxidant peptides through multiple mechanisms. Among these, the Keap1/Nrf2-antioxidant response element (ARE) signaling pathway is recognized as the most crucial regulator in the body’s endogenous defense against oxidative stress [5]. During oxidative stress, Keap-1 interacts with free radicals, causing it to detach from Nrf2. Once free, Nrf2 translocates into the nucleus, where it initiates the activation of the antioxidant response element (ARE) and drives the expression of genes responsible for producing antioxidant enzymes [39]. Peptides with antioxidant properties can influence the Keap1/Nrf2-ARE signaling cascade, thereby enhancing their antioxidant effects [52]. The results from the molecular docking analysis suggest that peptide residues in F < 3 kDa could interact directly with the active sites of Keap1, providing deeper insights into the antioxidant activity of these peptides. In this sense, some polypeptides identified from soft-shell turtles can enhance antioxidant response activity (ARE) by both upregulating Nrf2 and downregulating Keap1 [51]. In a different investigation, three antioxidant peptides extracted from the digestive fluid of snakehead fish successfully bound to the active sites of Keap1, forming more hydrogen bonds and π interactions than the control peptide TX6 [53].

**Figure 6:**
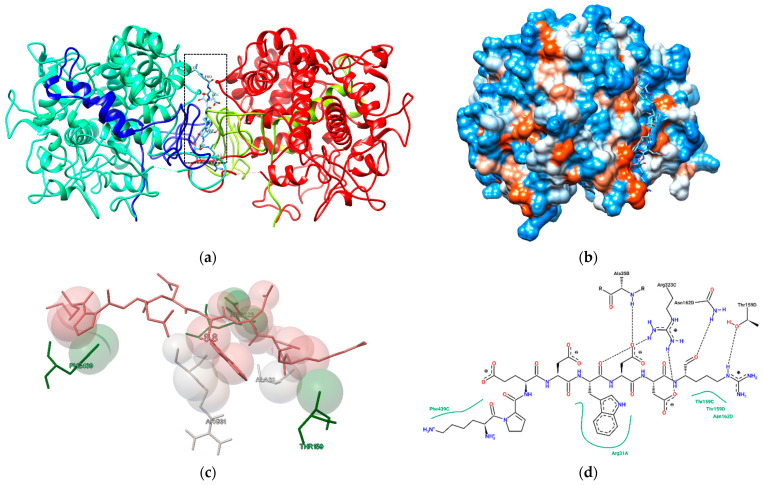
Molecular interaction of the KPEDWDDR peptide-MPO: (**a**) molecule in quaternary representation; (**b**) surface hydrophobicity diagram; (**c**) charge interactions (white color: MPO molecule; red color: KPEDWDDR peptide; green color: hydrogen bonds); (**d**) 2D interaction diagram.

Furthermore, Figure 6 illustrates the docking posture of the peptide with MPO. This enzyme interacts with H_2_O_2_ generated by phagocytic cells upon exposure to foreign particles, forming an enzyme–substrate complex that exhibits a potent oxidative capacity [3]. Increased MPO activity has been described in various pathological processes and is associated with an increased likelihood of developing diseases associated with oxidative stress, such as in infectious diseases (general or local), inflammatory diseases (rheumatoid arthritis), and ischemia/reperfusion [4]. The docking posture of the peptide with MPO is similar to the results reported for antioxidant peptides derived from sea cucumber, both of which blocked the entry to the MPO active cavity, confirming their effectiveness as antioxidants [49,54]. The interactions between MPO and the peptides involved hydrogen bonding and electrostatic forces (see Table 3). The peptide KPEDWDDR exhibited multiple interactions with myeloperoxidase, where several binding sites matched those found in previous studies of MPO interactions with antioxidant peptides derived from sea cucumber. These sites include Arg31, Ala35, Thr159, Asn162, and Arg323 in chain D [49,54]. A plausible explanation for the antioxidant activity of peptides lies in their binding to these sites for the antioxidant effects observed in the cell line by obstructing H_2_O_2_ access to the MPO-containing nucleus and thereby decreasing ROS levels.

## 4. Conclusions

The hydrolysate of the red Californian worm and its low molecular weight fraction (F < 3 kDa) do not exhibit any cytotoxic effects and do not alter the redox state, cell cycle distribution, or cell death in differentiated Caco-2 cells. Additionally, the F < 3 kDa protects the cells from oxidative stress induced by hydrogen peroxide, maintaining cell viability and proper cell cycle progression. It also significantly maintains correct redox status (ROS, GSH, and intracellular calcium), and reduces the percentage of cells undergoing apoptosis and necrosis. The cytoprotective effect of F < 3 kDa peptides, with affinities for antioxidant molecules such as MPO and Keap1, against oxidative stress begins with a decrease in intracellular calcium, which results in a reduction in reactive oxygen species due to the inhibition of MPO. Added to this is the increase in GSH, due to the increase in Nrf-2, when the peptides bind to the inhibitory region (Keap-1) of this mediator and consequently the maintenance of the redox state, resulting in the correct distribution of the cell cycle and a decrease in the number of cells in the sub-G1 phase, indicating a decrease in early and late apoptosis. These findings highlight the potential application of WH as a natural source of bioactive peptides with antioxidant properties in the context of oxidative stress environments. Considering future research prospects there is a need to evaluate the peptides in in vivo assays and determine their cytoprotective effect directly.

## Figures and Tables

**Table 1:** The effect of worm hydrolysate (WH) and its fractions on the cytotoxicity in differentiated Caco-2 cells.

	Cell Viability (% of Control)		
Concentration (mg/mL)	WH	F > 3 kDa	F < 3 kDa
1	135.6 ± 6.6	158.5 ± 4.8	151.5 ± 4.3
0.5	113.5 ± 3.3	118.3 ± 9.1	131.0 ± 11.1
0.1	149.1 ± 4.7	143.8 ± 4.0	125.8 ± 8.7
0.075	139.3 ± 6.8	122.3 ± 7.0	103.6 ± 7.5
0.05	143.6 ± 4.3	148.2 ± 7.4	116.1 ± 7.7
0.025	130.5 ± 15.1	105.6 ± 7.2	108.4 ± 7.0

**Table 2:** Peptides from the fraction < 3 kDa identified by LC-MS/MS using the Tims TOF-fleX.

Peptides	Molecular Weight (Da)	Peptide Size	Score	Source Protein
SLLDDRLDEK	1203.62	10	0.909	Hemoglobin linker chain
KPEDWDDR	1060.47	8	0.9454	Calreticulin
DDDDDGDGIPDSK	1362.51	13	1	Ofus.G011730 protein
EKKEEDKDKPK	1372.72	11	1	HSP83 protein
KRVGPGLGEY	1075.59	10	0.9237	Ferritin

**Table 3:** Affinity energies and interactions of the worm hydrolysate (WH) peptides with antioxidant molecules (KEAP-1 and MPO).

Sequence	Kelch-like ECH-Associated Protein 1			Myeloperoxidase		
	Affinity Energy (Kcal/mol)	Interactions	Hydrogen Bonds	Affinity Energy (Kcal/mol)	Interactions	Hydrogen Bonds
DDDDDGDGIPDSK	−6.4			−7.4	Thr159; Ile160; Ala28; Phe29; Pro34; Val30; Arg31; Asp321; Trp32; Lys505;	Arg323; Aal35;
EKKEEDKDKPK	−5.5	Arn483; Tyr525; Gly509; Gln530; Ala556; Tyr572; Ser602; Phe577; Asn382;	Tyr334; Asn414; Arg415;	−7.0	Ile180; Arg31; Ala35; Pro34; Trp32; Lys505; Phe439;	Cys153; Asn162; Arg31;
KPEDWDDR	−7.3	Gly509; His436; Phe478; Ser508; Tyr525; Ser555; Ala556; Arg483; Gly603; Phe577; Asn382; Gly462;	Arg380; Asn382; Asn414; Arg415;	−8.8	Arg31; Ala28; Ala35; Phe439; Thr159; Pro151; Arg151	Ala35; Arg323; Asn162;
KRVGPGLGEY	−6.9	Arg415; Ser508; Tyr525; Ser555; Phe577; Ala556; Ser602; Gln530; Tyr572; Gly433; Asp389;	Arg380; Asn387; Arg415; Arg483;	−7.9	Asn162; Ile160; Arg31; Ala35	Arg323; Lys505;
SLLDDRLDEK	−7.3	Arg483; His436; Ser431; Gly452; Ser508; Tyr525; Ser555; Ser363; Gly364; Tyr572; Tyr334	Arg380; Asn414; Arg415; Gln530;	−7.3	Thr159; Arg31; Arg323; Phe429;	Ile160;

## Data Availability

The original contributions presented in the study are included in the article, further inquiries can be directed to the corresponding author.

## Supplementary Materials

The following supporting information can be downloaded at: https://www.mdpi.com/article/10.3390/nu16213654/s1, Figure S1: The mass spectra of peptides identified by LC-MS/MS from the <3 kDa fraction.
