# Supplementary material for: Cytoprotective Effects of Antioxidant Peptides from Red Californian Worm (Eisenia foetida) Hydrolysate on Differentiated Caco-2 Cells
Source: Nutrients. 2024 Oct 27;16(21):3654. doi: 10.3390/nu16213654 (PMC11547318; doi:10.3390/nu16213654)
Supplement: Supplementary file 1 [file nutrients-16-03654-s001.zip › nutrients-3261534-supplementary.pdf]

Figure S1: The mass spectra of peptides identified by LC-MS/MS from the <3 kDa fraction.

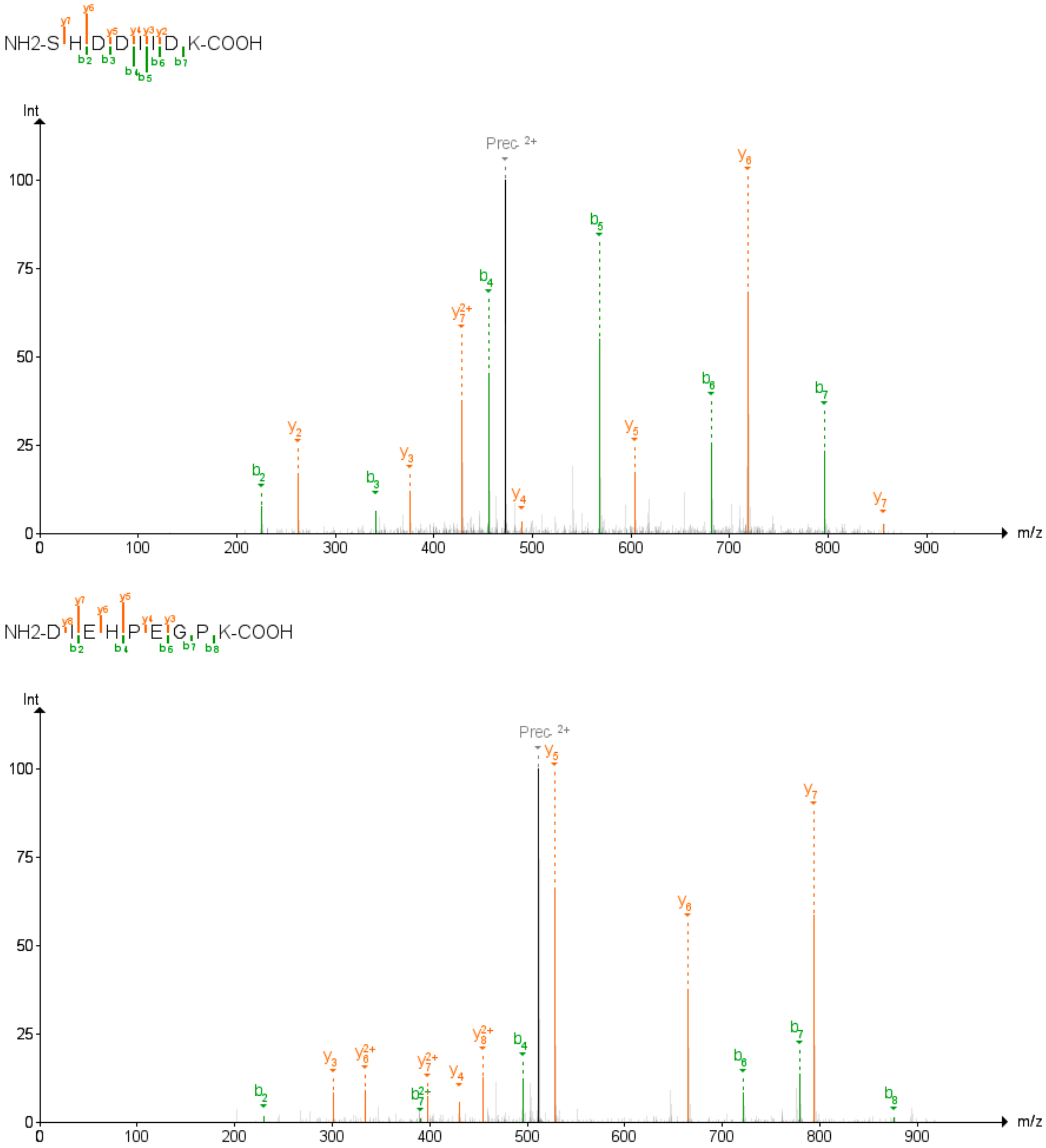

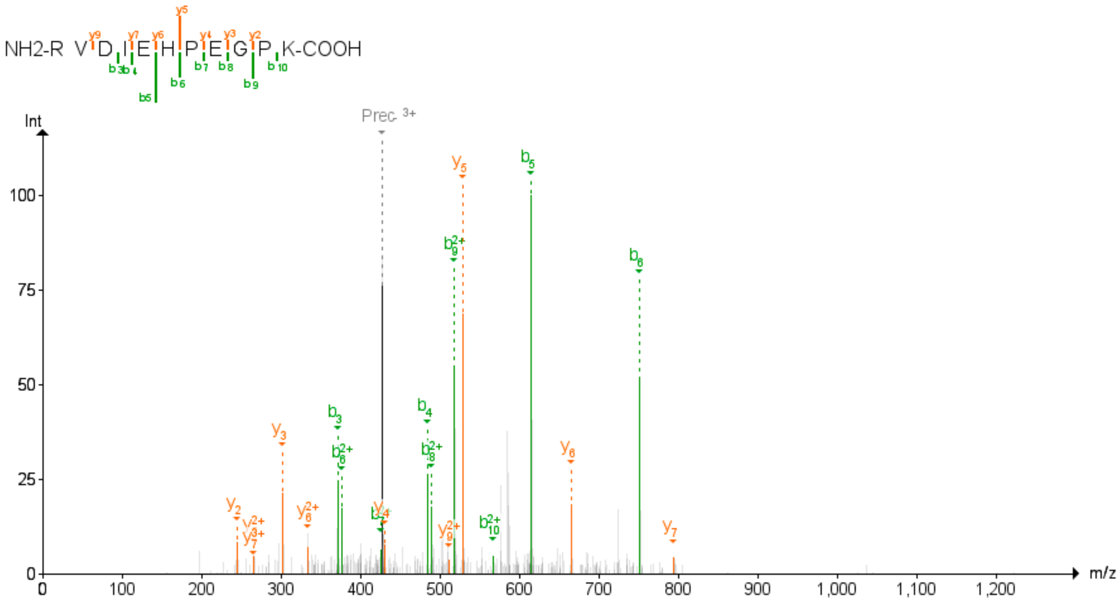
NH2-R V D I E H P E G P K-COOH
Int
m/z
Prec 3+
100
75
50
25
0
0
100
200
300
400
500
600
700
800
900
1,000
1,100
1,200

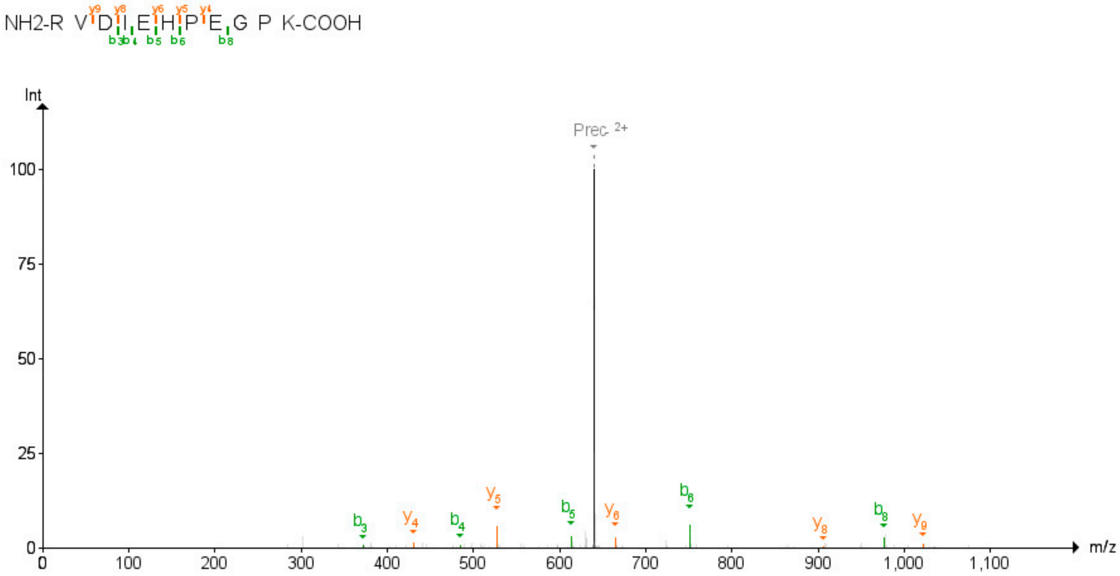
NH2-R V D I E H P E G P K-COOH
Int
m/z
Prec 2+
100
75
50
25
0
0
100
200
300
400
500
600
700
800
900
1,000
1,100

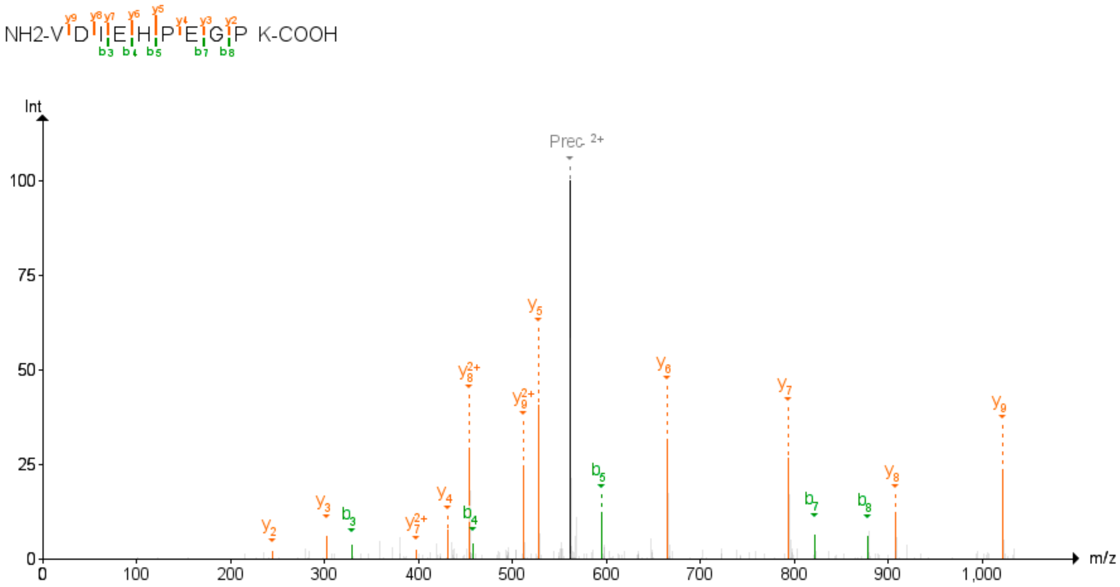
NH2-V D I E H P E G P K-COOH
Int
Prec 2+
m/z
0
25
50
75
100
100
200
300
400
500
600
700
800
900
1,000

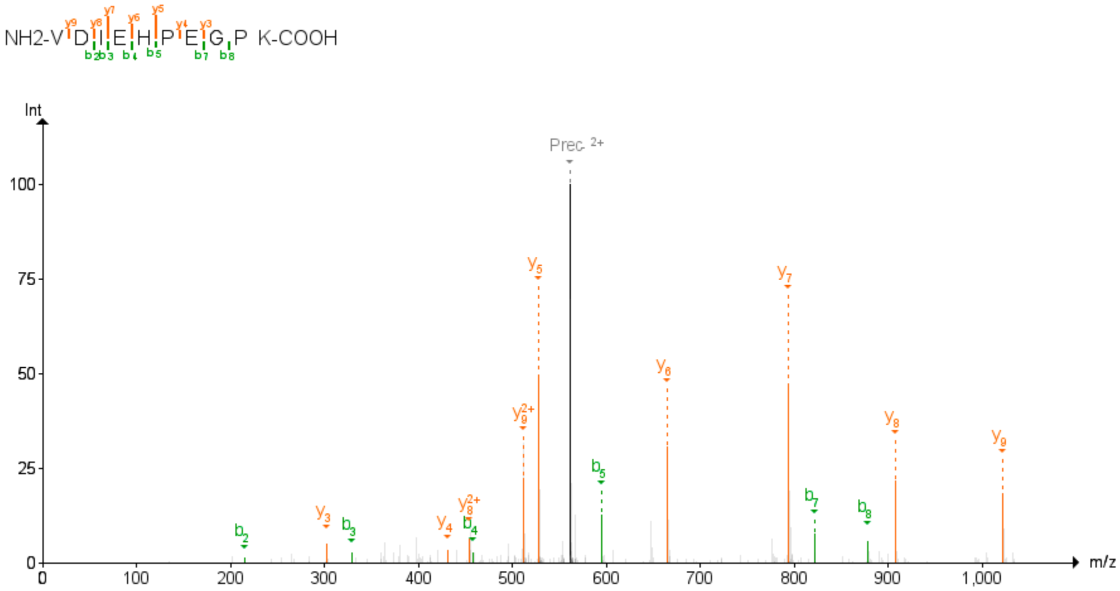
NH2-V D I E H P E G P K-COOH
Int
Prec 2+
m/z
0
25
50
75
100
100
200
300
400
500
600
700
800
900
1,000

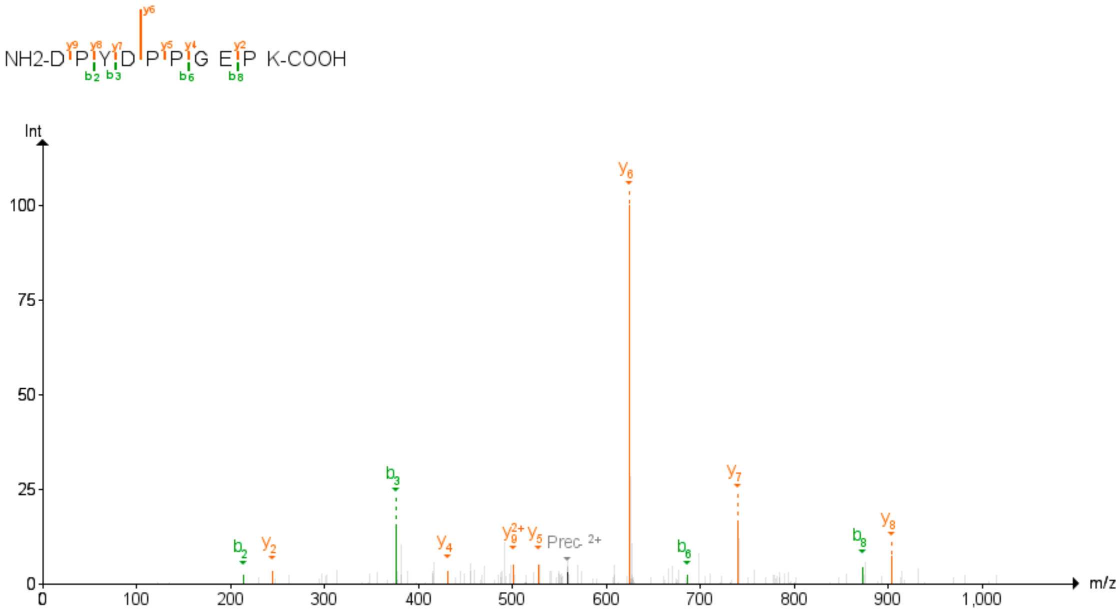

NH2-D P Y D P P G E P K-COOH
Int
m/z
Prec 2+

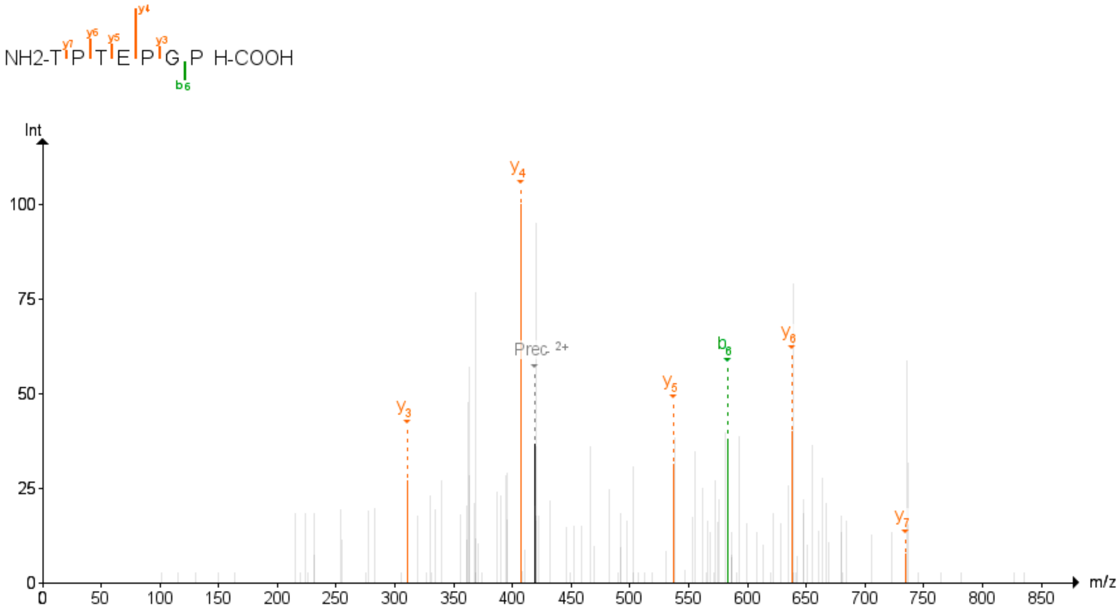

NH2-T P T E P G P H-COOH
Int
m/z
Prec 2+

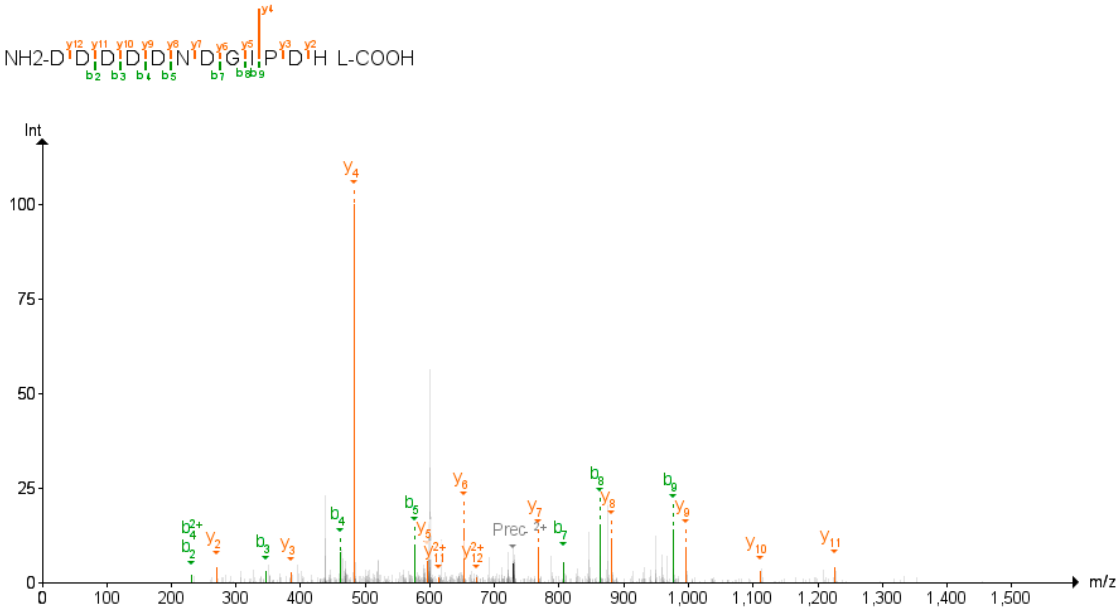

NH2-D D D D D N D G I P D H L-COOH
Int
m/z
Prec 2+

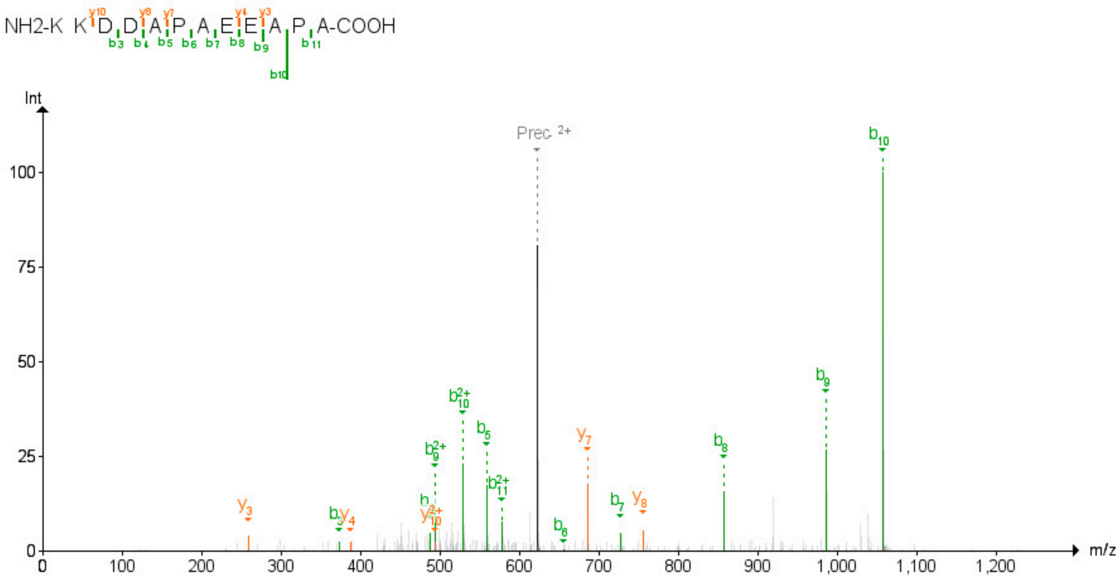

NH2-K K D D A P A E E A P A-COOH
Int
m/z
Prec 2+

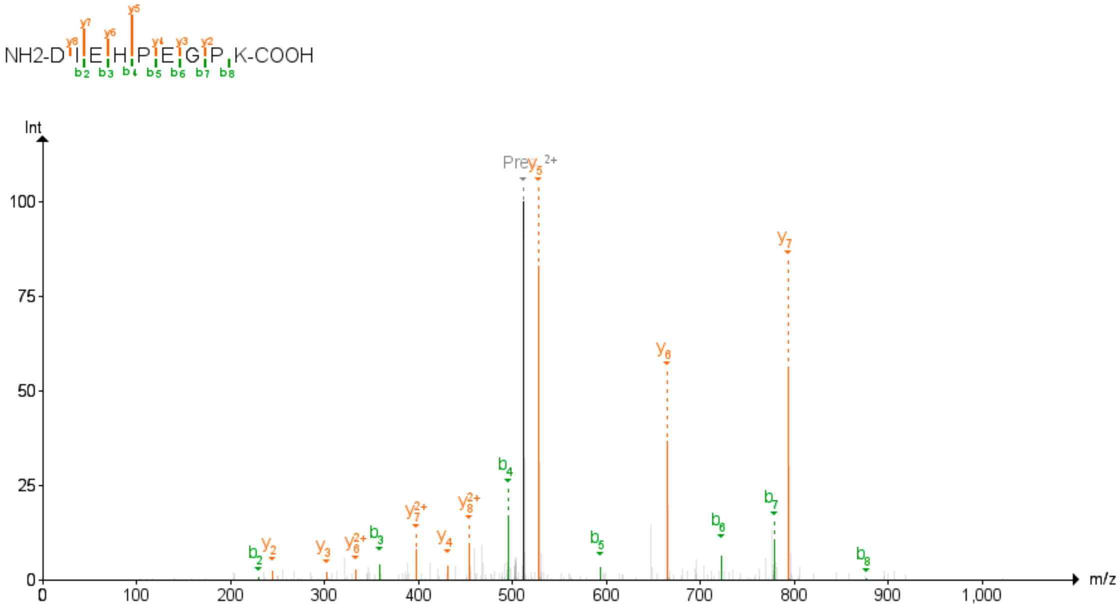
NH2-D I E H P E G P K-COOH
Int
Prec
y5 2+
y7
y6
b4
b7
b6
y8 2+
y7 2+
b5
y4
y6 2+
b3
y3
b2
y2
b8
100
75
50
25
0
0
100
200
300
400
500
600
700
800
900
1,000
m/z

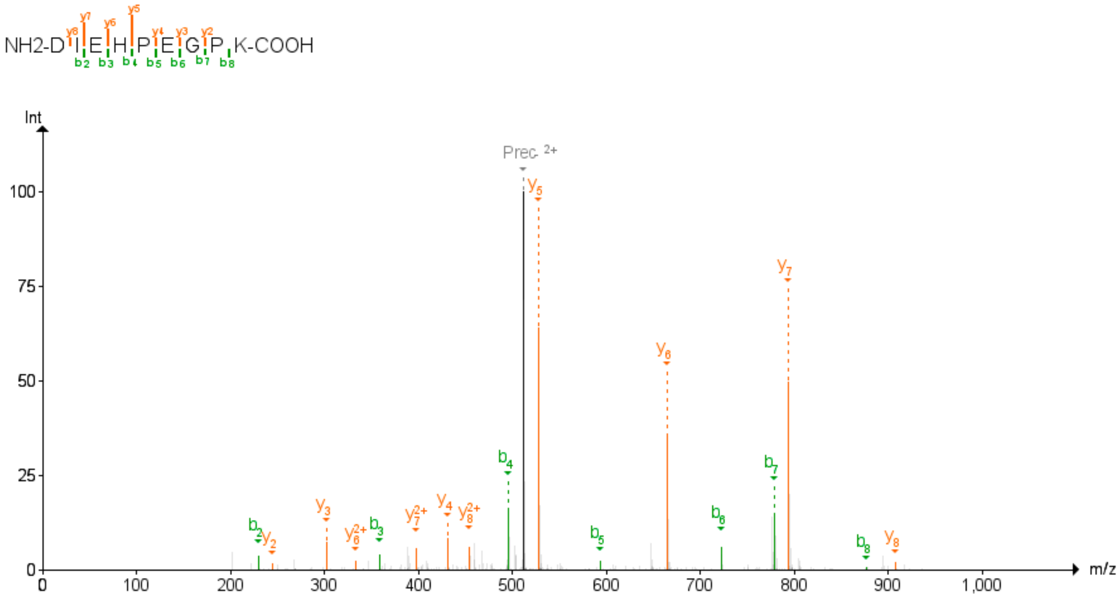
NH2-D I E H P E G P K-COOH
Int
Prec 2+
y5
y7
y6
b4
b7
b6
y3
y4
y8 2+
y7 2+
b2
y6 2+
b3
b5
y2
b8
y8
100
75
50
25
0
0
100
200
300
400
500
600
700
800
900
1,000
m/z

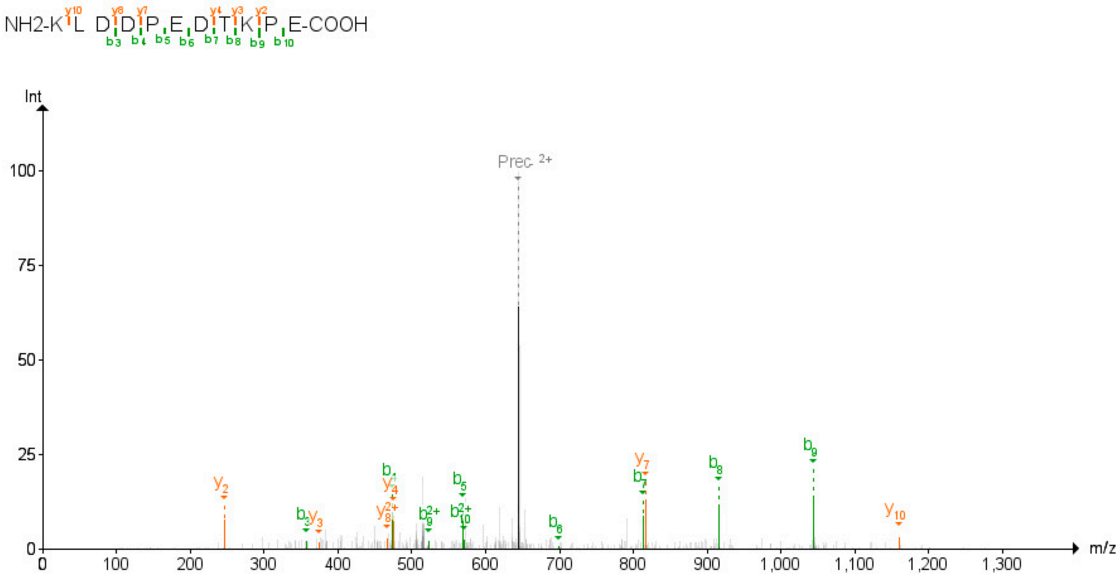
NH2-KLDDPEDTKPE-COOH
Int
Prec. 2+
m/z

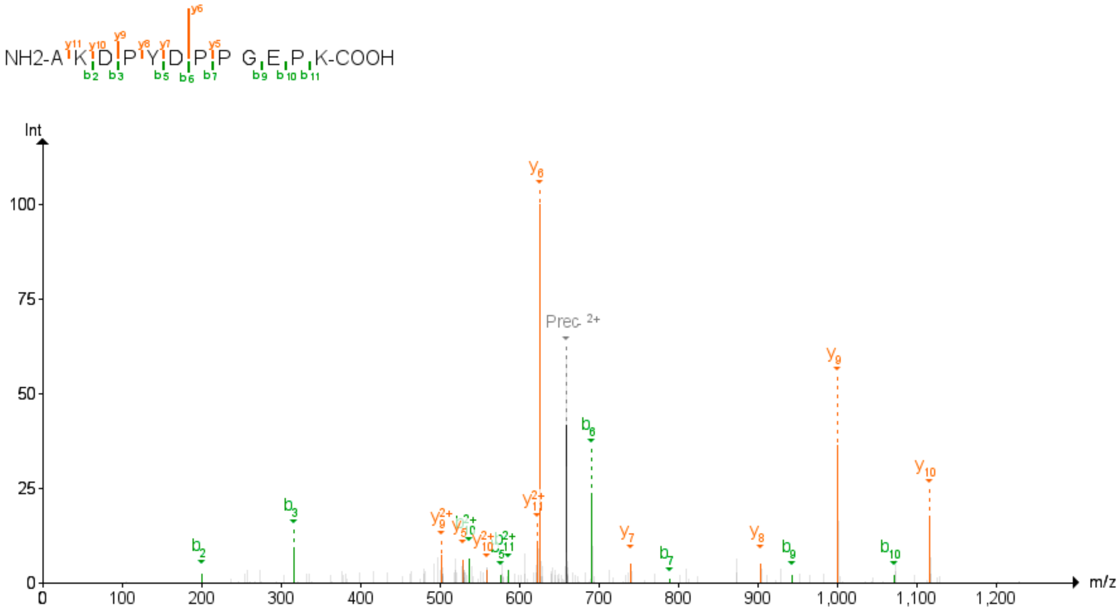
NH2-AKDPYDPPGEPK-COOH
Int
Prec. 2+
m/z

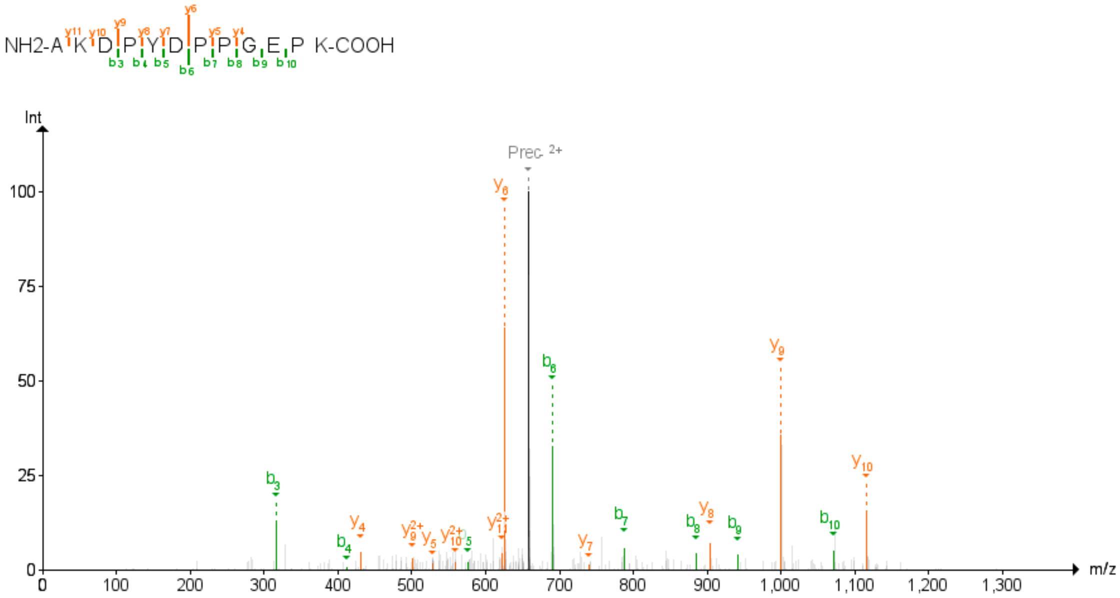
NH2-AKDPYDPPGEP K-COOH
Int
Prec 2+
m/z

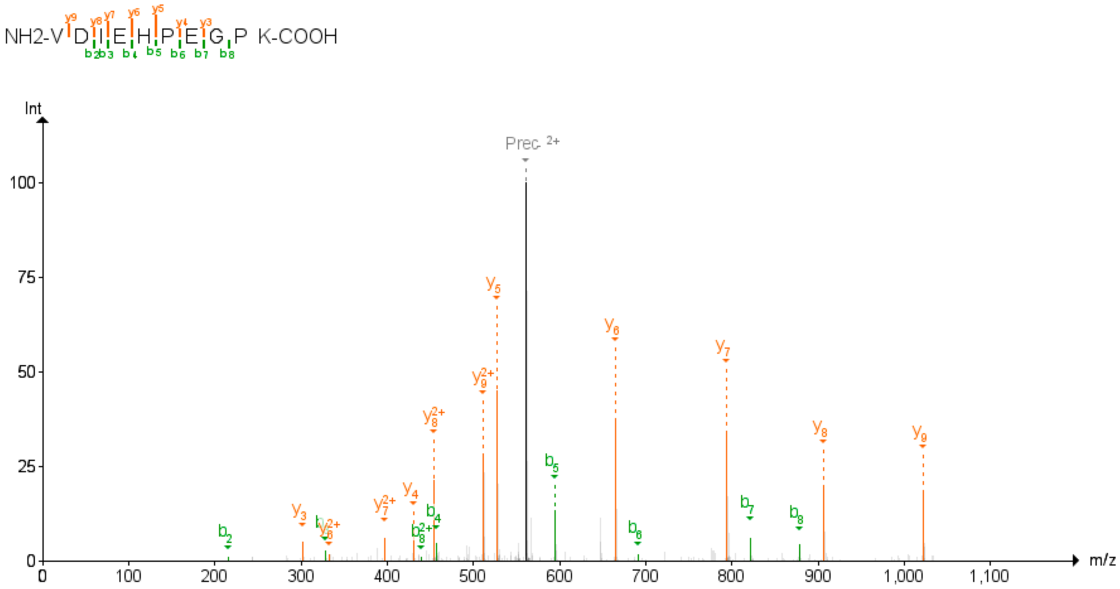
NH2-VDIEHPEGP K-COOH
Int
Prec 2+
m/z

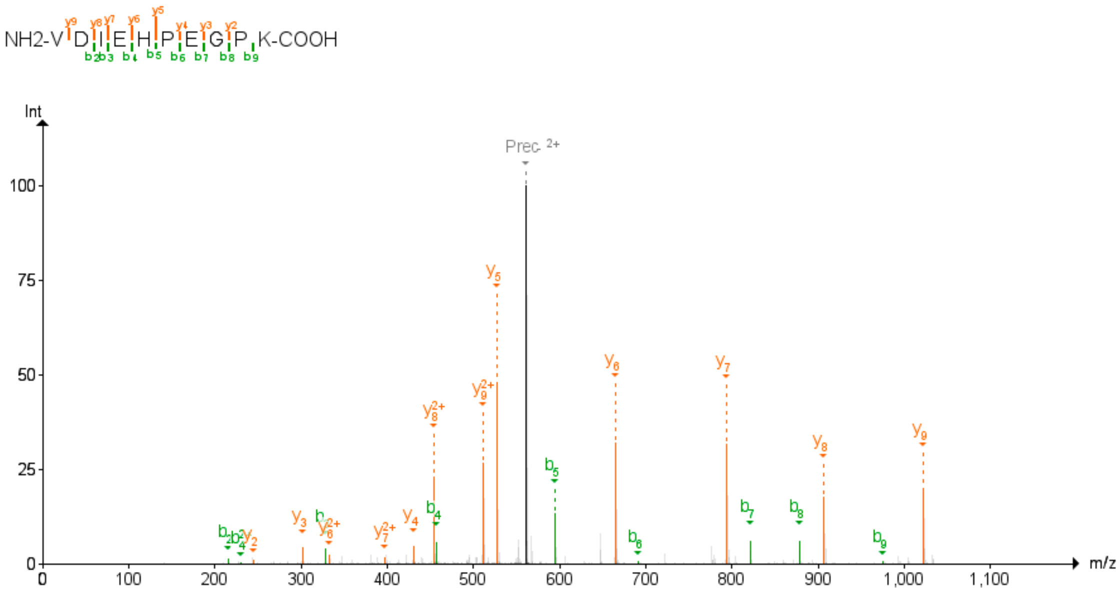
NH2-V D I E H P E G P K-COOH
Int
Prec 2+
m/z
100
75
50
25
0
0
100
200
300
400
500
600
700
800
900
1,000
1,100

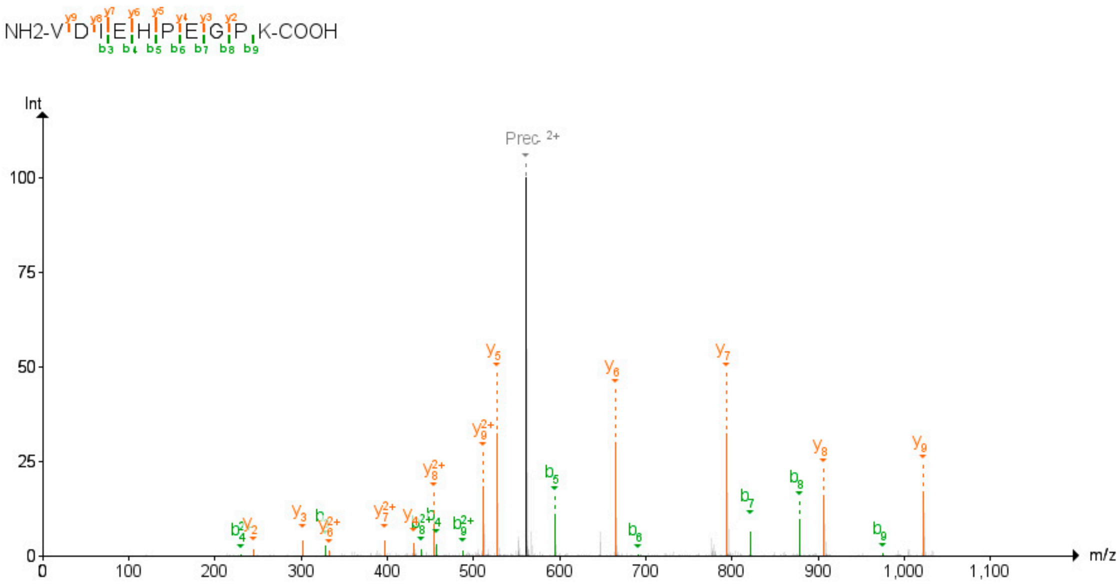
NH2-V D I E H P E G P K-COOH
Int
Prec 2+
m/z
100
75
50
25
0
0
100
200
300
400
500
600
700
800
900
1,000
1,100

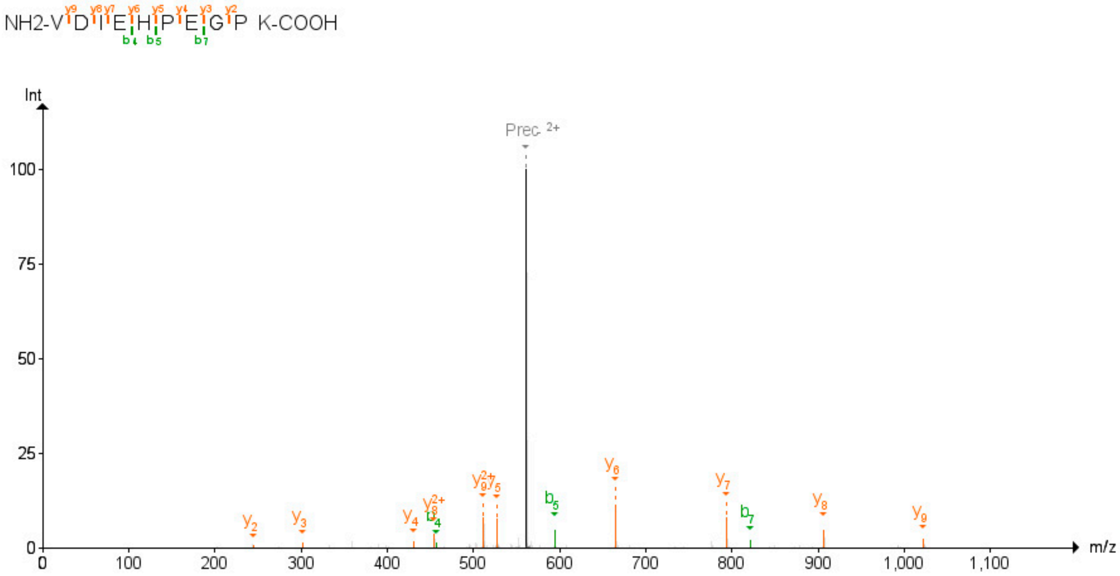
NH2-V D I E H P E G P K-COOH
Int
Prec 2+
100
75
50
25
0
m/z
0
100
200
300
400
500
600
700
800
900
1,000
1,100

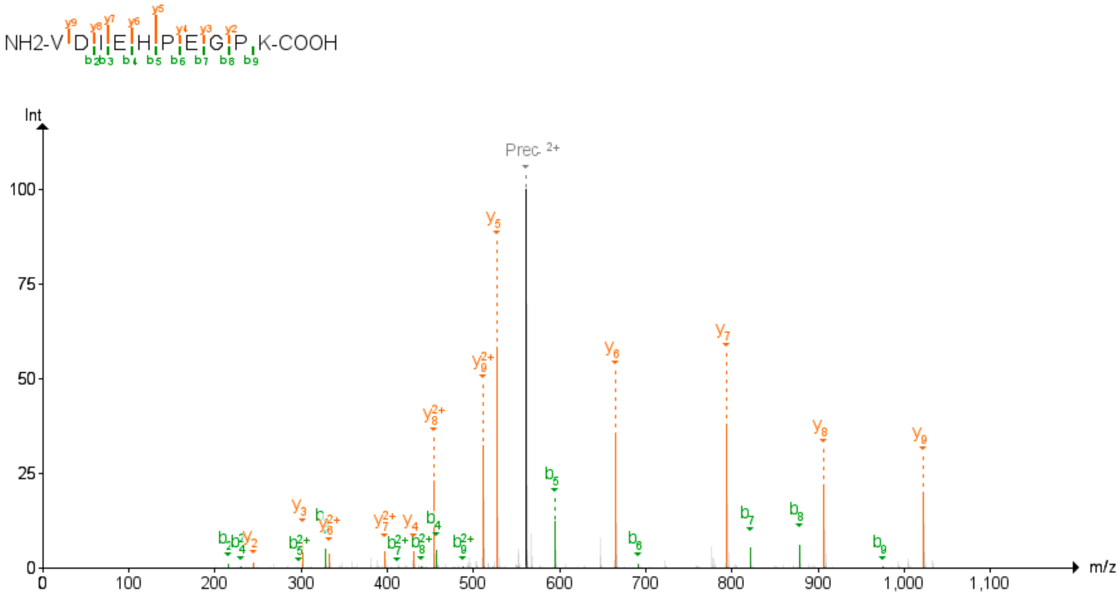
NH2-V D I E H P E G P K-COOH
Int
Prec 2+
100
75
50
25
0
m/z
0
100
200
300
400
500
600
700
800
900
1,000
1,100

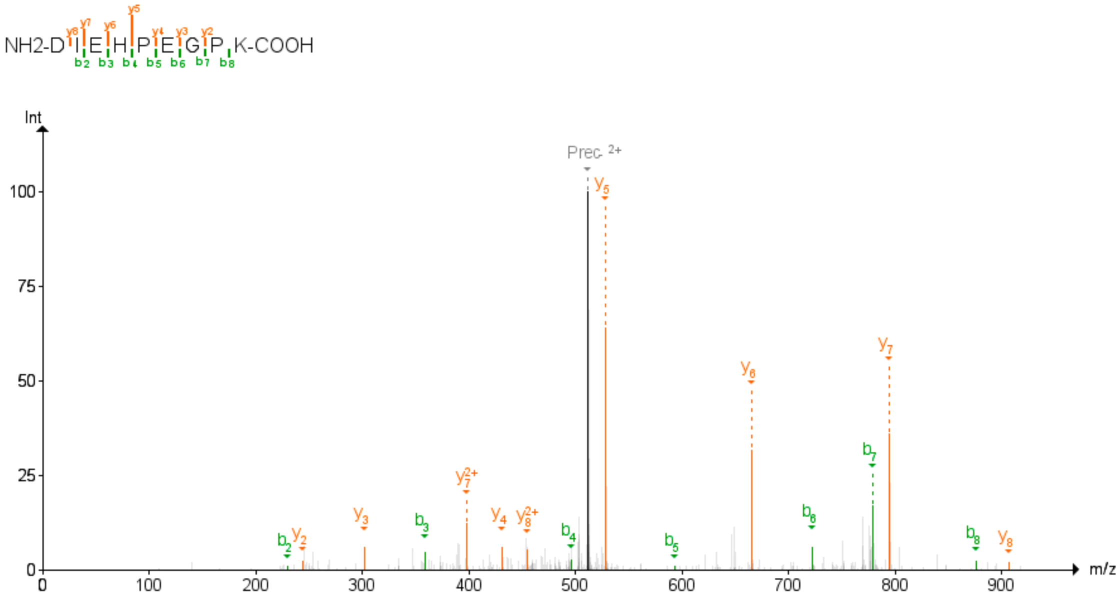
NH2-D I E H P E G P K-COOH
Int
Prec 2+
m/z

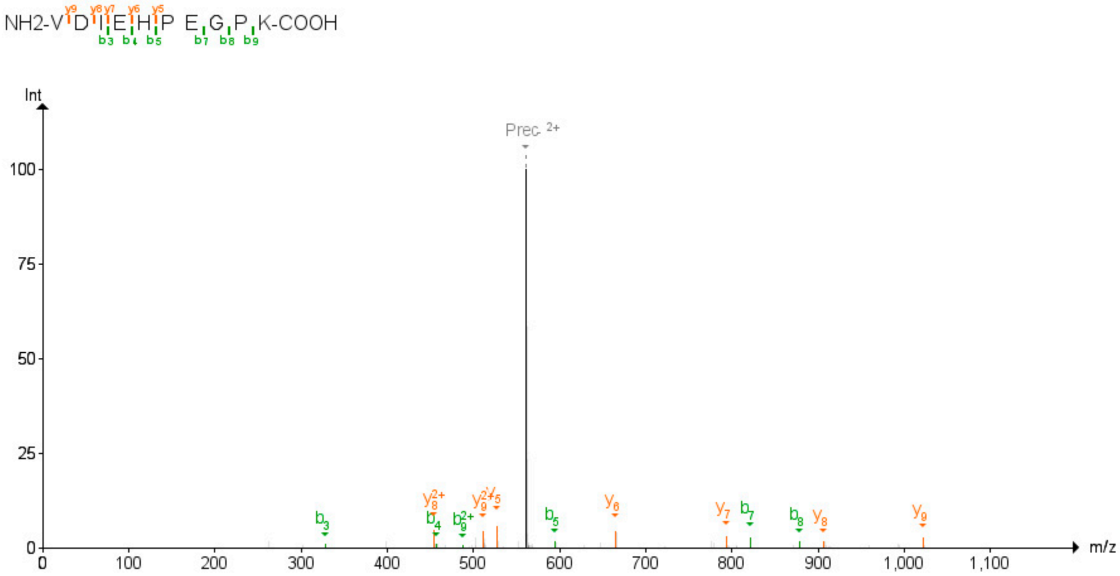
NH2-V D I E H P E G P K-COOH
Int
Prec 2+
m/z

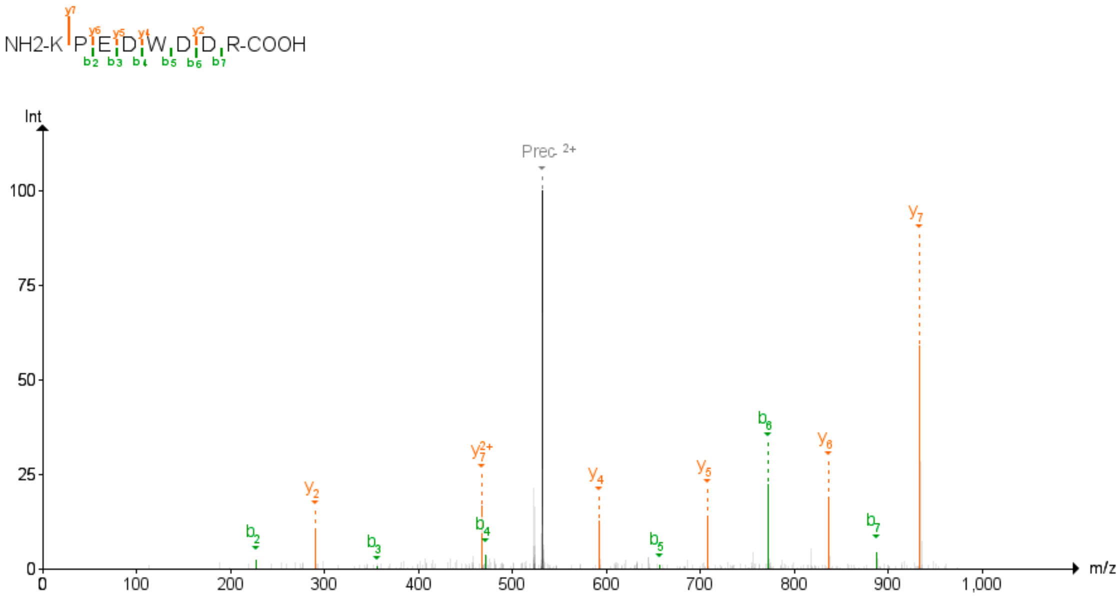
NH2-K P E D W D D R-COOH
Int
Prec 2+
100
75
50
25
0
0
100
200
300
400
500
600
700
800
900
1,000
m/z

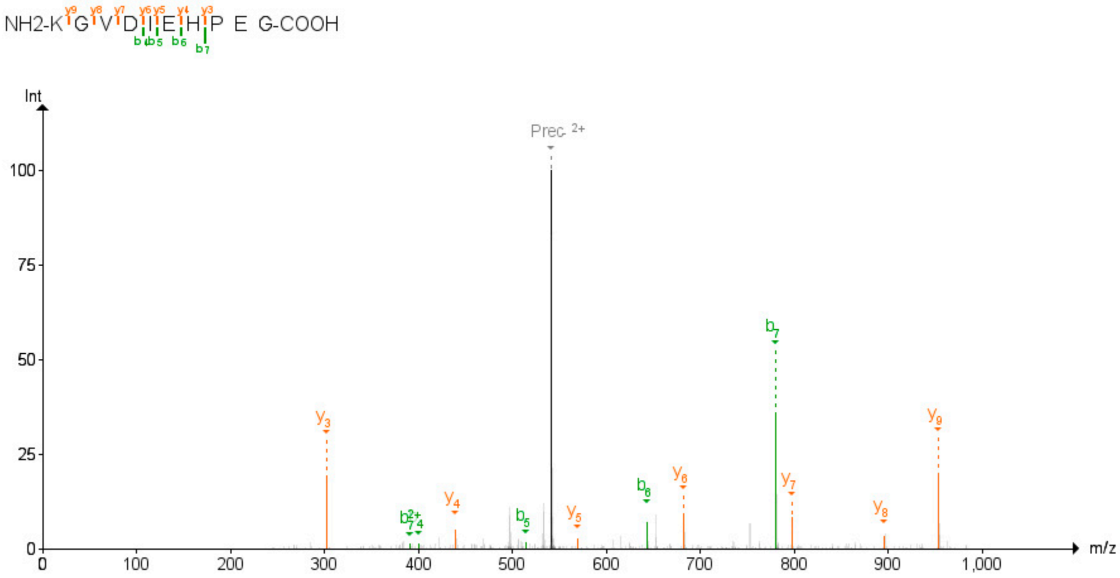
NH2-K G V D I I E H P E G-COOH
Int
Prec 2+
100
75
50
25
0
0
100
200
300
400
500
600
700
800
900
1,000
m/z

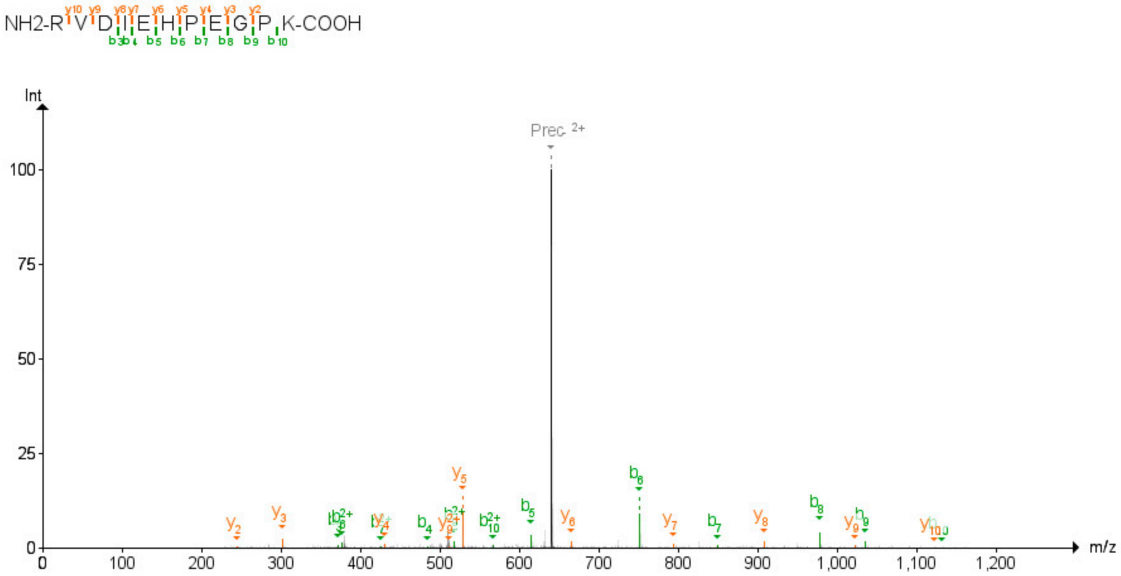
NH2-RVDIIEHPEGPK-COOH
Int
Prec 2+
m/z

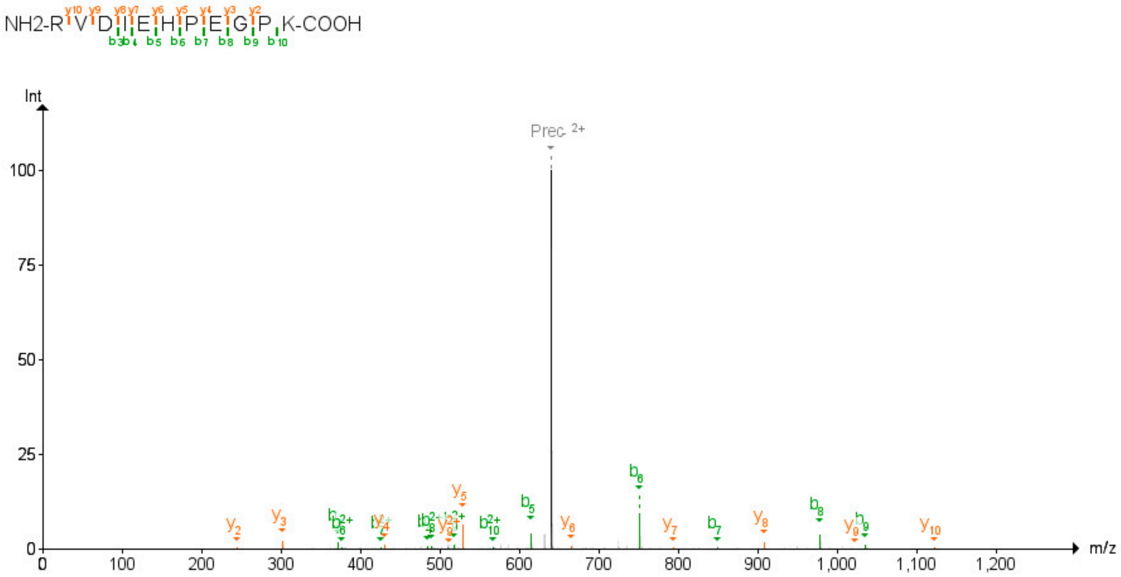
NH2-RVDIIEHPEGPK-COOH
Int
Prec 2+
m/z

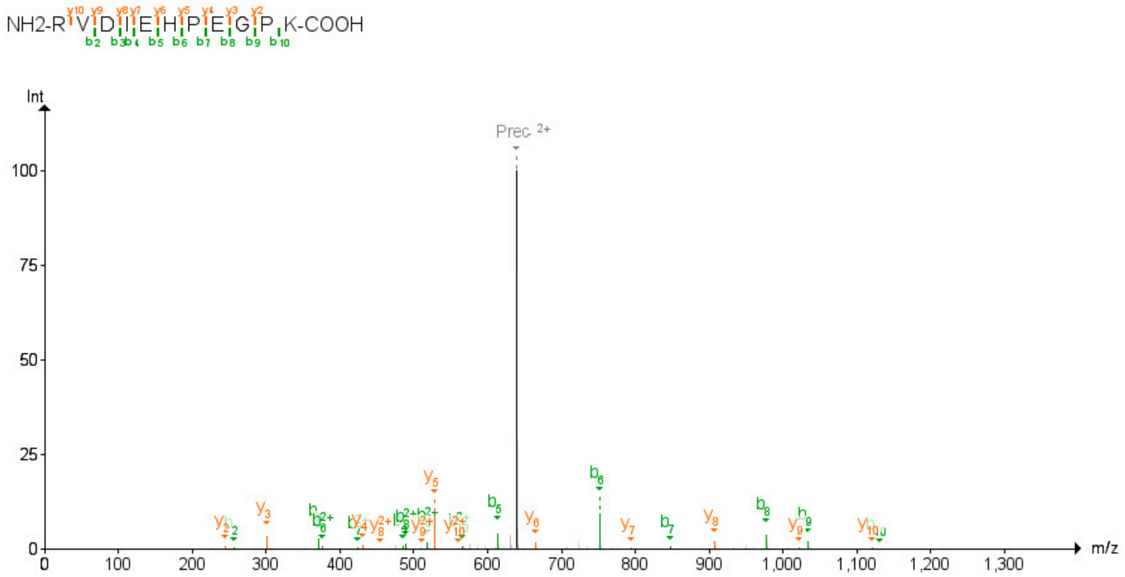
NH2-RVDIIEHPEGPK-COOH
Int
Prec 2+
m/z

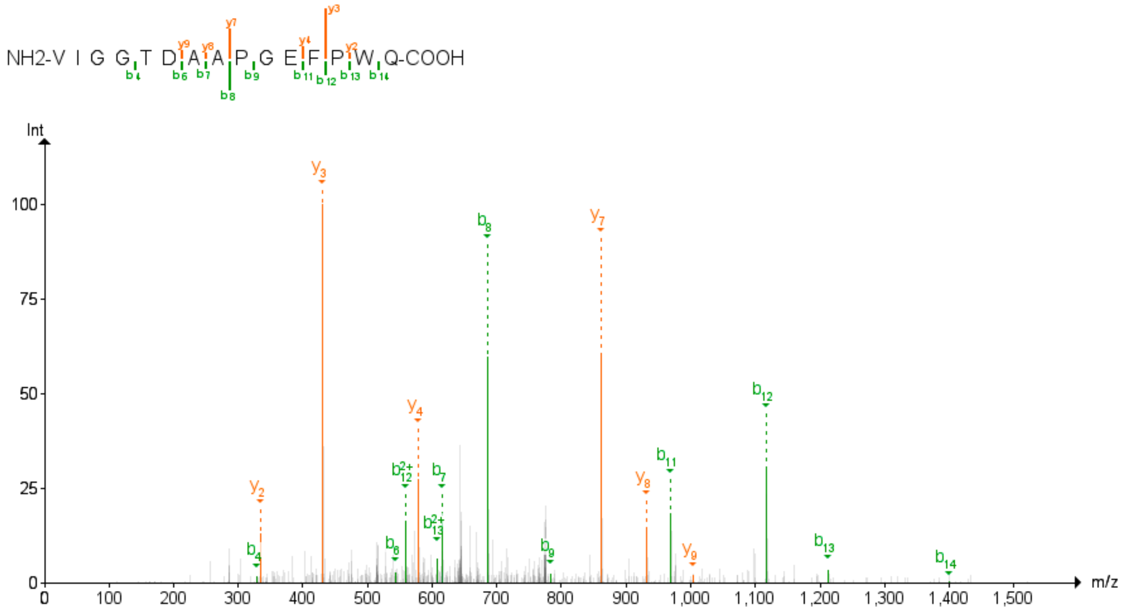
NH2-VIGGTDAAPGEFPWQ-COOH
Int
m/z

NH2-RVDIIEHPEGPK-COOH

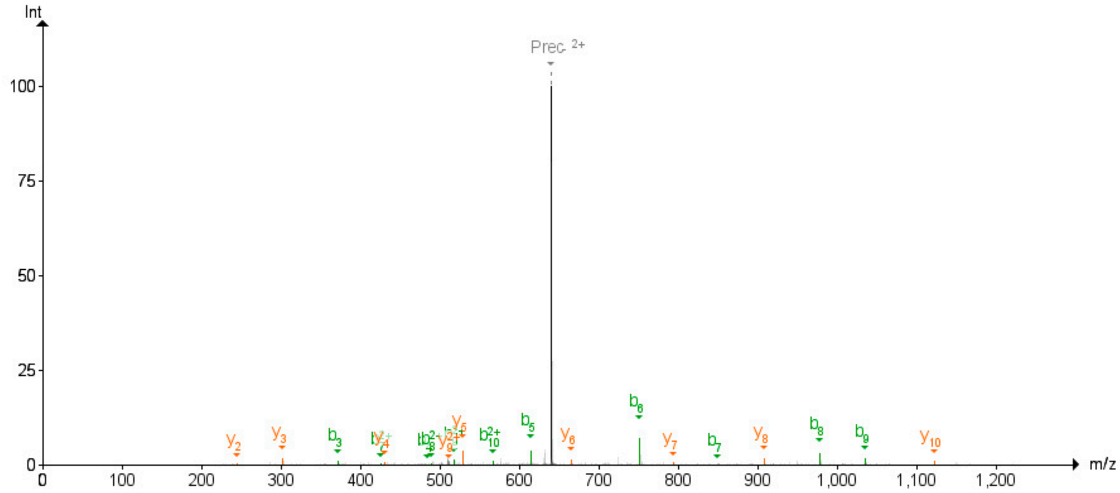

NH2-KRVGPGLGEY-COOH

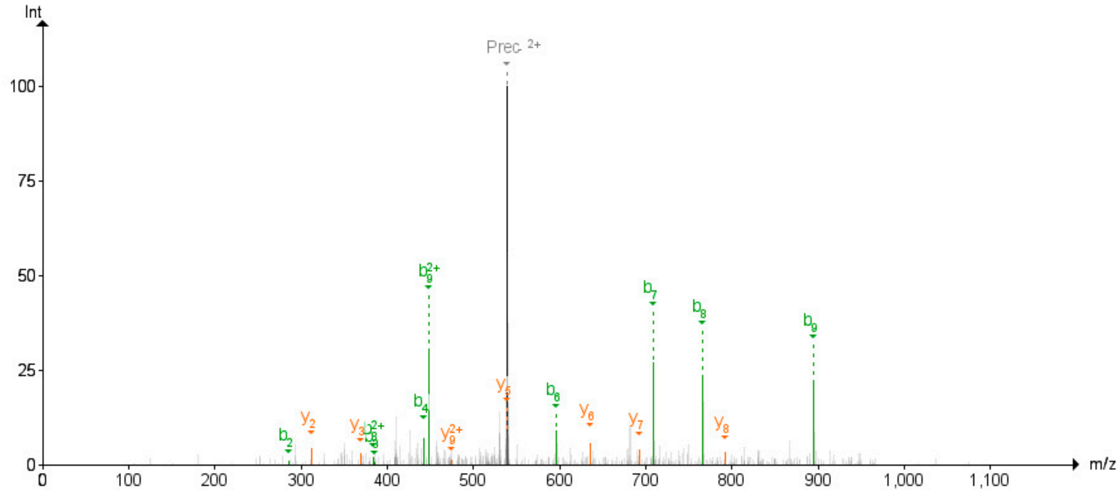

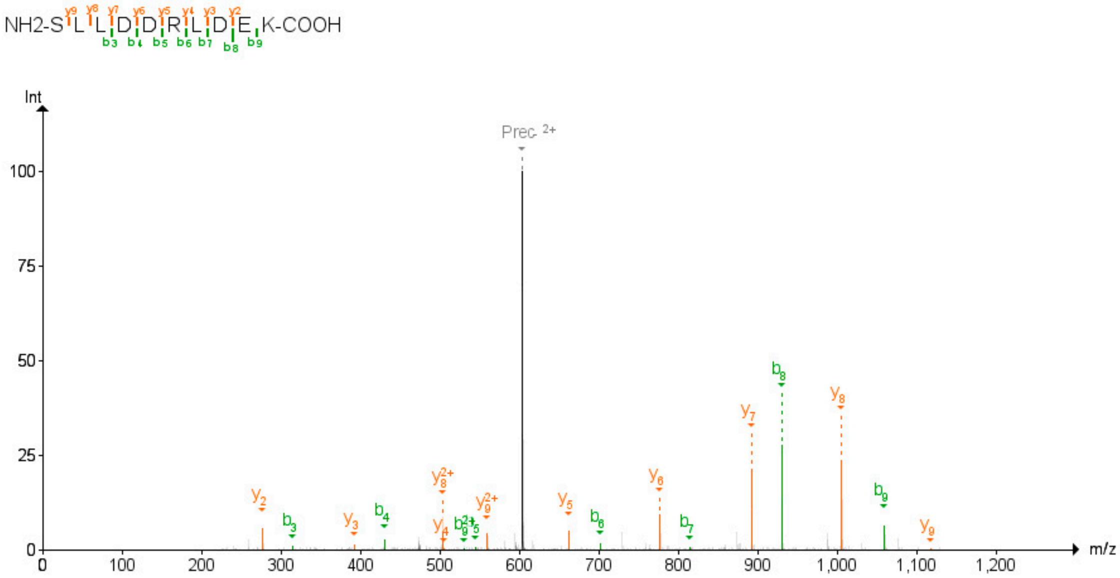
NH2-SLLDDRLDEK-COOH
Int
Prec 2+
m/z

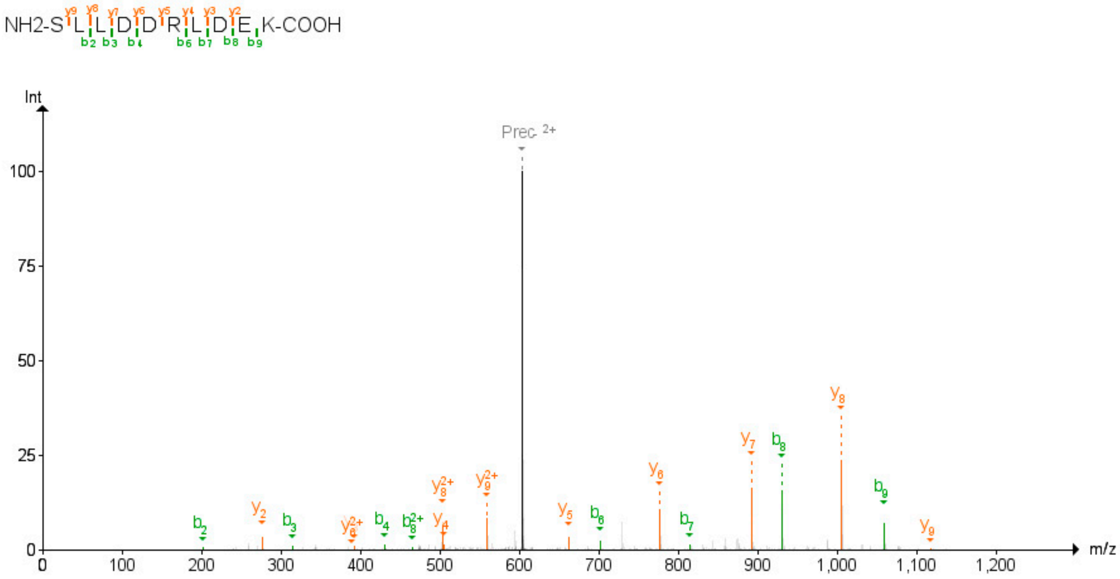
NH2-SLLDDRLDEK-COOH
Int
Prec 2+
m/z

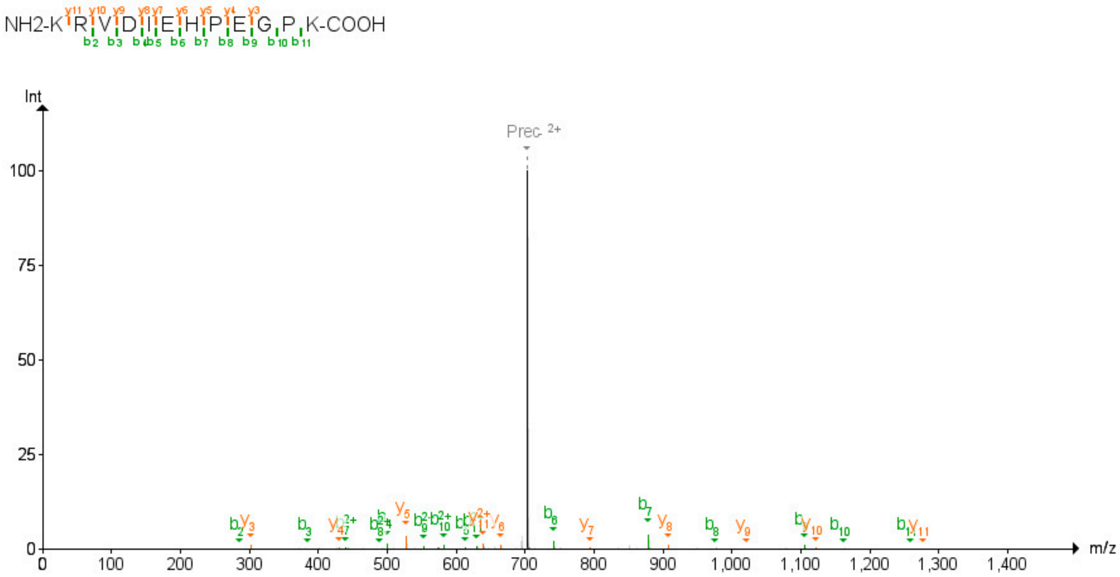

NH2-K R V D I I E H P E G P K-COOH
Int
Prec 2+
m/z
0
25
50
75
100
0
100
200
300
400
500
600
700
800
900
1,000
1,100
1,200
1,300
1,400

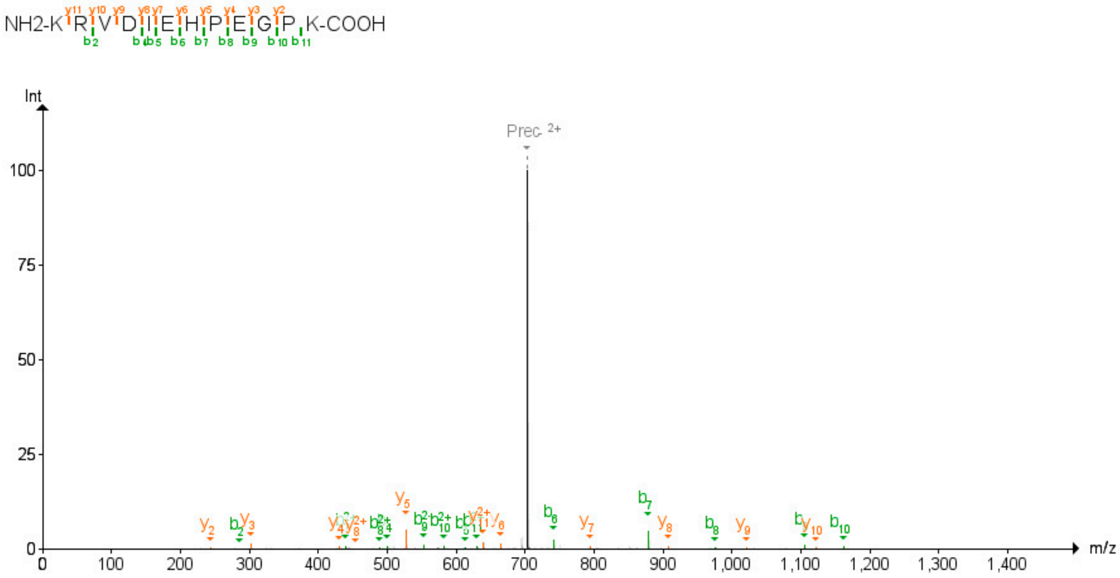

NH2-K R V D I I E H P E G P K-COOH
Int
Prec 2+
m/z
0
25
50
75
100
0
100
200
300
400
500
600
700
800
900
1,000
1,100
1,200
1,300
1,400

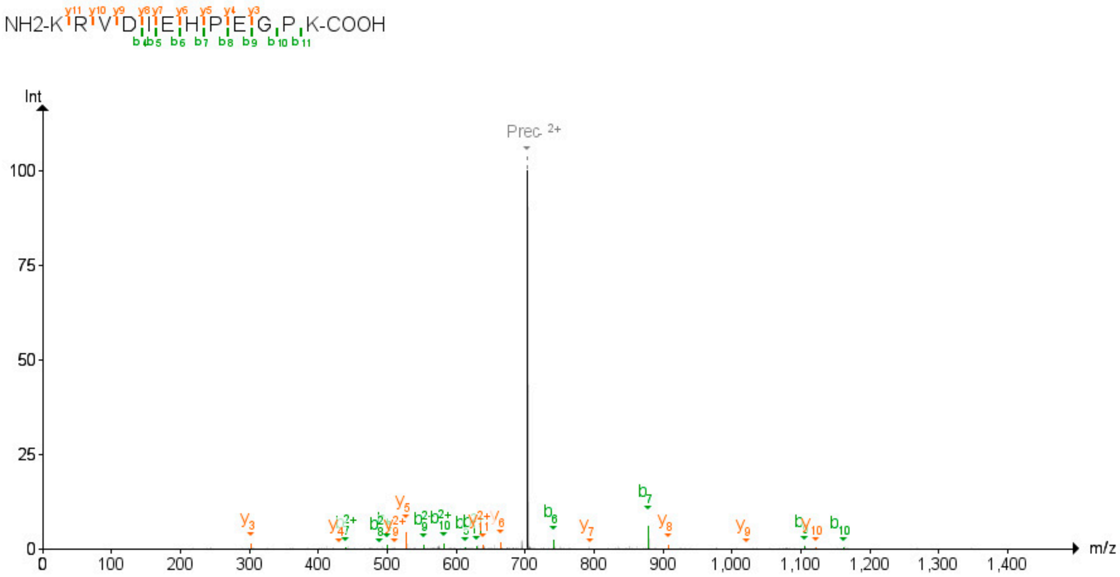
NH2-K R V D I I E H P E G P K-COOH
Int
Prec. 2+
m/z
0
25
50
75
100
0
100
200
300
400
500
600
700
800
900
1,000
1,100
1,200
1,300
1,400

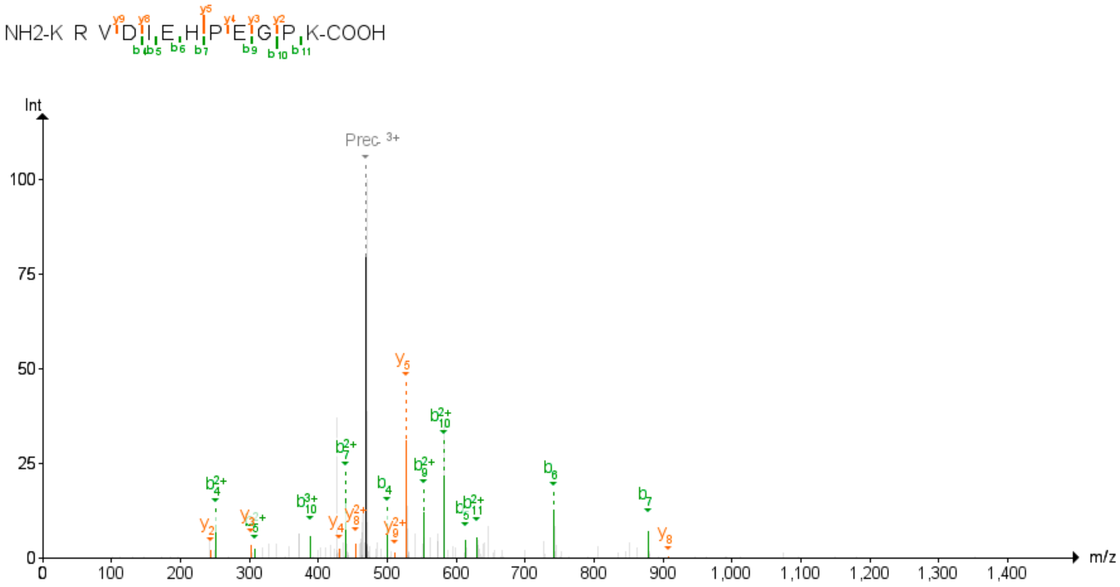
NH2-K R V D I I E H P E G P K-COOH
Int
Prec. 3+
m/z
0
25
50
75
100
0
100
200
300
400
500
600
700
800
900
1,000
1,100
1,200
1,300
1,400

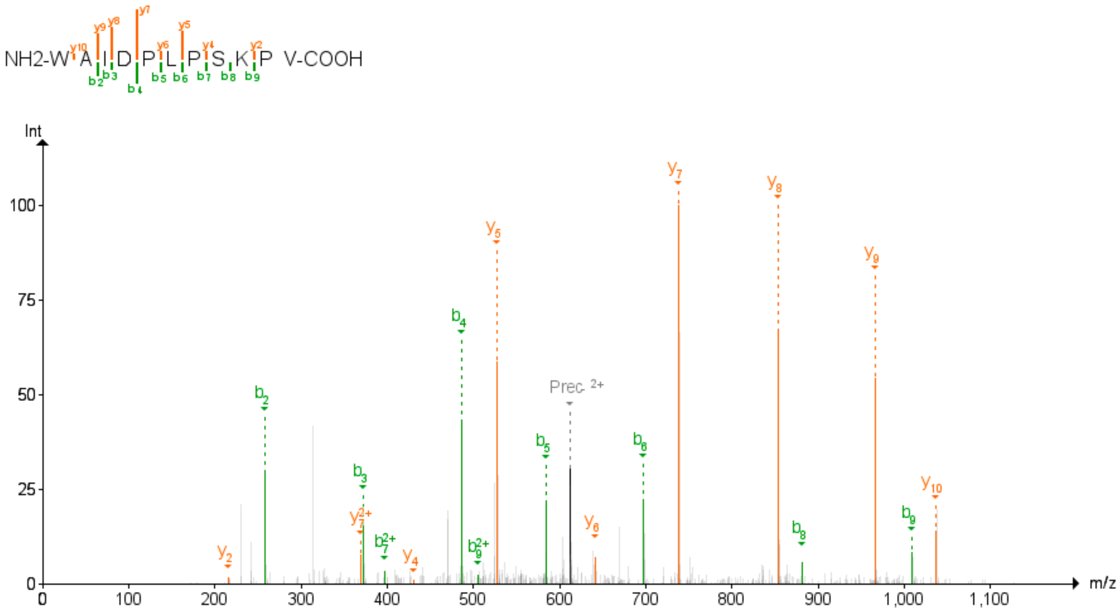
NH2-W A I D P L P S K P V-COOH
Int
m/z
Prec 2+

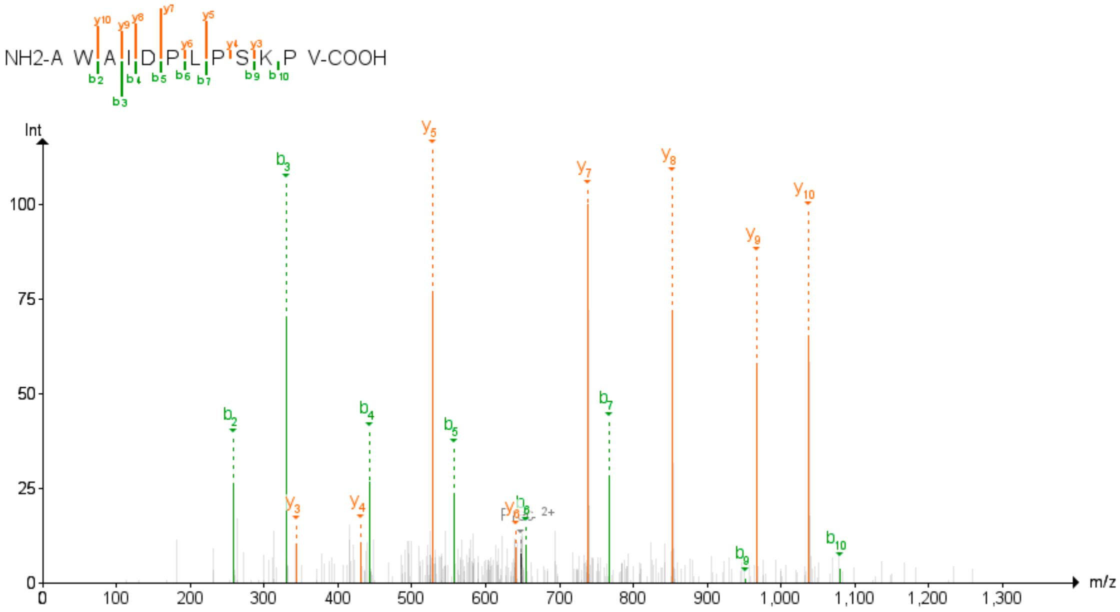
NH2-A W A I D P L P S K P V-COOH
Int
m/z

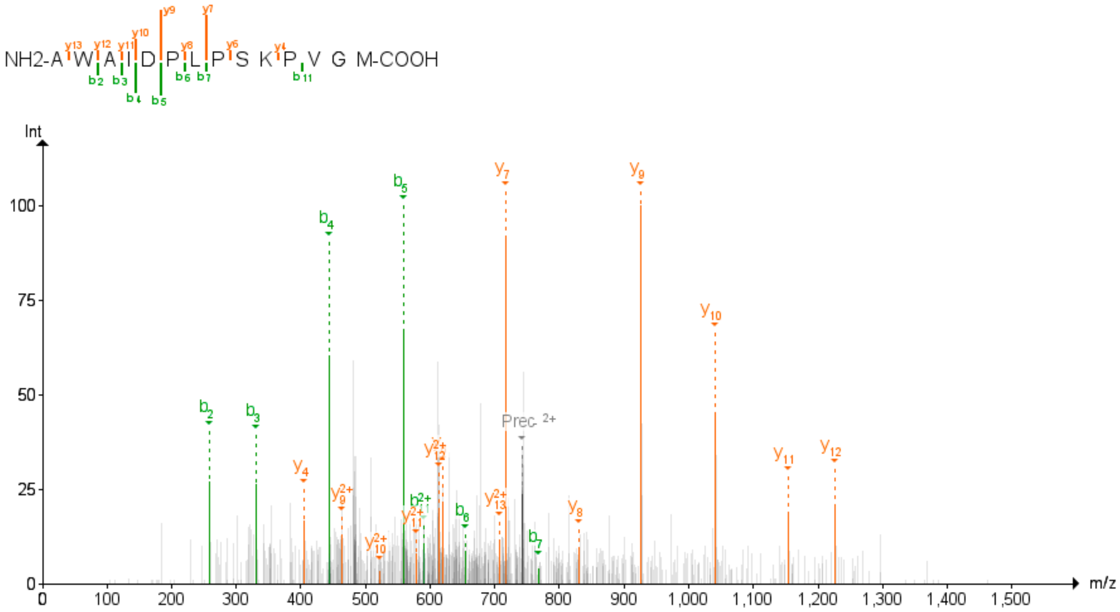

NH2-A W A I D P L P S K P V G M-COOH
Int
m/z
Prec 2+
